# Structural quality-tier assessment for TCR-pMHC functional enrichment

**DOI:** 10.3389/fimmu.2026.1869810

**Published:** 2026-07-24

**Authors:** Alex Ascunce-París, Miguel Romero-Durana, Alfonso Valencia, Roc Farriol-Duran, Víctor Guallar

**Affiliations:** 1 Barcelona Supercomputing Center - Centro Nacional de Supercomputación (BSC-CNS), Plaça d’Eusebi Güell, Barcelona, Spain; 2 Faculty of Pharmacy and Food Sciences, University of Barcelona (UB), Barcelona, Spain; 3 Catalan Institution for Research and Advanced Studies (ICREA), Pg. Lluís Companys, Barcelona, Spain; 4 Cancer Genome Evolution Research Group, University College London Cancer Institute, London, United Kingdom; 5 Cancer Research UK Lung Cancer Centre of Excellence, University College London Cancer Institute, London, United Kingdom

**Keywords:** AlphaFold3, protein modelling, structural quality assessment, T cell receptor, T-cell immunotherapies, TCR-pMHC specificity

## Abstract

T cell receptor (TCR) recognition of peptide-MHC complexes (pMHCs) is central to adaptive immunity. Structural insights into TCR-pMHC interactions are critical for understanding antigen specificity and T-cell function. However, progress remains limited by the scarcity of experimentally resolved structures (275 TCR-pMHC class I structures in the PDB, Jan 2026). Although protein structure modelling tools have advanced rapidly, accurate structural modelling of TCRs remains challenging due to CDR loop hypervariability and conformational flexibility. In addition, there is a lack of reliable quality assessment strategies that do not rely on comparisons with experimental references. To address this, we benchmarked four general-purpose (AlphaFold2.3-Multimer, AlphaFold3, Boltz-2, Chai-1) and three TCR-specific (TCRmodel2, tFold-TCR, TCRdock) protein modelling algorithms by recalculating all experimentally determined TCR-pMHC class I complexes in the PDB. AlphaFold3 demonstrated superior performance across metrics (mean TCR-iRMSD = 3.59 Å and DockQ = 0.54), whereas the other algorithms displayed lower accuracy. Built on AlphaFold3 structures, we present a scalable and interpretable ML framework for the quality assessment of TCR-pMHC structural models without matched experimental references. We trained a random forest classifier integrating multiple confidence metrics (pLDDT, ipTM, ipSAE, iPAE, iPDE, pDockQv1-2) derived from 1325 modelled structures of 265 experimentally determined PDB TCR-pMHC class I complexes. The classifier reliably stratifies structural models into low-, acceptable-, medium-, and high-quality tiers defined by comparisons to their experimental reference structures, outperforming single metrics. These quality-tier predictions further enable the prioritization of high-confidence TCR-pMHC interactions. This was demonstrated across two held-out datasets comprising a total of 4,090 AlphaFold3-modelled TCR-pMHC complexes (20,450 models, 5 models per complex): a re-evaluated set of TCR-pMHC class I complexes from VDJdb (n = 606) and the reference IMMREP23 dataset (n = 3,484). We profiled the first dataset to reduce false-positive TCR-pMHC interactions erroneously annotated in VDJdb, and the second to enrich for biologically validated TCR-pMHC interactions amongst higher-quality structural models over their synthetic negative counterparts. Altogether, our structural quality-tier framework provides a scalable and interpretable approach that complements structural modelling and functional analyses of TCR-pMHC class I complexes, with direct translational applications in T-cell immunology and TCR-based immunotherapies.

## 1 Introduction

Recognition of peptide-MHC complexes (pMHCs) by T cell receptors (TCRs) is a central event in adaptive cellular immunity (1). Most TCRs are αβ heterodimers that mediate antigen recognition through six hypervariable loops, known as complementarity-determining regions (CDRs). Each chain contains three CDRs, which together interact with the presented peptide or epitope and/or the MHC molecule (2). To maximize coverage of potential epitopes, human TCR repertoires are generated by V(D)J recombination, a somatic gene rearrangement process that combines variable (V), diversity (D), and joining (J) gene segments with random nucleotide insertions and deletions at the junctions. This mechanism is estimated to generate over 10¹¹ distinct receptors in an individual, out of approximately 10^15^ potential possibilities, prior to thymic selection. As a result, each individual is equipped with extraordinary clonal diversity in their TCR repertoire (3, 4). This diversity fosters T cell responses to a vast array of foreign antigens, including pathogens and cancer-associated peptides, while maintaining tolerance to self.

Structural insights into TCRs and their cognate pMHC partners are essential for understanding the geometry and biophysics of the TCR-pMHC interface, which is governed by recognition specificity, and ultimately drives T-cell activation (5, 6). However, the experimental determination of TCR-pMHC structures remains challenging (7, 8), with only a few hundred TCR-pMHC class I complexes deposited in the PDB (9) (n=275, date Jan 2026). Recent advances in protein structure prediction have opened new opportunities for modelling TCR-pMHC complexes using existing sequence datasets with annotated experimental validation. The utility of these structural models has been demonstrated across a range of applications, supporting structural characterization and providing mechanistic insights into T-cell recognition (10–20). Protein structure prediction algorithms have evolved substantially beyond traditional homology-based approaches (21–24), particularly following the breakthroughs achieved by AlphaFold (25–28). Examples include general-purpose tools such as AlphaFold-Multimer v2.3 (28), AlphaFold3 (25), Chai-1 (26), Boltz-2 (27), as well as others trained or tuned specifically for TCRs, such as TCRmodel2 (29), TCRdock (12) or tFold-TCR (30). Although these methods can predict molecular complexes with remarkable accuracy, immune receptors such as antibodies and TCRs remain particularly challenging targets. The extreme sequence variability and conformational flexibility of CDR loops, combined with the limited number of experimentally determined structures, pose major challenges for immune receptor structural prediction (31, 32).

Despite advances in immune receptor modeling, assessing the quality of predicted TCR-pMHC models remains a major challenge. Unlike benchmark studies where experimental structures are available (33, 34), practical applications require evaluating model reliability in the absence of structural references. In this context, quality assessment is particularly difficult, as the most robust evaluation metrics rely on structural comparisons with matched experimentally determined complexes. Several confidence metrics that can be derived directly from predicted models have been proposed, including pLDDT, ipTM, iPAE (35), iPDE (36), ipSAE (37) and pDockQ (v1 and v2) (38, 39). However, their performance in immune complexes remains limited (55, 56).

Beyond structural modeling challenges, recent deep-learning and AI-based algorithms have attempted to predict TCR-pMHC pairing directly from sequence data (40–44). Nonetheless, most approaches fail to generalize predictions to unseen epitopes, defined as individual peptides not presented in any TCR-pMHC complex included in the training set of a given tool (45–48). Importantly, the predictive accuracy of specificity predictors heavily relies on the annotation quality of TCR-pMHC complexes in sequence databases such as VDJdb (49). A recent re-validation effort of VDJdb has reported a substantial fraction of false positives or interactions of uncertain confidence, among two immunodominant epitopes: GLCTLVAML (Epstein-Barr virus) and YLQPRTFLL (SARS-CoV-2), each paired with multiple TCRs and restricted to HLA-A*02:01. This has led to the creation of the TCR validated database (TCRvdb) (50), which provides a curated collection of higher-confidence TCR-pMHC triads. Alternatively, we posit that integrating biophysical information derived from structural data could help capture binding motifs that govern molecular recognition and ultimately could improve the prediction of novel, unseen epitopes (10–16, 51–53). This further underscore the need for rigorous structural model quality assessment to ensure that only reliable predictions are used in downstream applications.

Given all these challenges, in this study, we benchmarked state-of-the-art general-purpose and TCR-specific protein modeling tools on experimentally determined TCR-pMHC class I complexes deposited in the PDB (9) comprising 265 TCR-pMHC class I structures. This allowed us to identify the best-performing approaches for TCR-pMHC modeling. Building on these results, we developed a novel, explainable ML framework to assess structural model quality, enabling the filtering of low-quality models and the retention of high-confidence structures. While trained on experimentally determined TCR-pMHC complexes, the framework is designed to evaluate new predicted models without requiring an experimental counterpart. Furthermore, we demonstrate that structural quality stratification can help to identify meaningful TCR-pMHC interactions. We applied this framework to thousands of predicted TCR-pMHC structural models generated from sequences annotated in TCRvdb (49, 50) and IMMREP23 (46). The IMMREP23 dataset was generated for a Kaggle competition on TCR-pMHC specificity predictors. It includes 3,484 class I complexes spanning 20 epitopes and captures broader biological diversity than TCRvdb. It is inherently imbalanced, with approximately a 1:5 ratio of positive to negative TCR-pMHC triads, reflecting a more realistic large-scale TCR screening scenario. Our analysis revealed clear distinctions between high- and low-confidence models across both datasets, helping to separate meaningful from less reliable TCR-pMHC interactions.

The proposed framework provides a unified strategy for evaluating and categorizing TCR-pMHC class I structural models in the absence of experimental references. Moreover, the results suggest a link between structural confidence and functional relevance. Overall, this study provides a tool for selecting more reliable TCR-pMHC structures, supporting downstream immunological analyses and serving as a complementary tool for translational applications in T-cell- and TCR-based immunotherapies (54).

## 2 Results

### 2.1 Benchmark of protein modelling tools reveals AlphaFold3’s superior performance on TCR-pMHC class I complexes beyond its training data

We evaluated several state-of-the-art (SOTA) structure prediction tools using the sequences of experimentally resolved TCR-pMHC class I complexes retrieved from the PDB (9). We excluded non-canonical docking poses involving inverted geometries or non-classical constant-domain-driven interfaces, as well as complexes from species other than mouse or human, resulting in a dataset of 265 TCR-pMHC complexes. We included four general-purpose algorithms: AlphaFold-Multimer v2.3 (28) (AF2.3), AlphaFold3 (25) (AF3), Chai-1 (26), Boltz-2 (27) and three TCR-specific methods TCRmodel2 (29), tFold-TCR (30) and TCRdock (12). Our benchmark set included two groups of TCR-pMHC complexes: those deposited before (pre) the training cutoff dates of each tool and those deposited after (post). For comparability, we used the most recent cutoff date (Boltz-2: 2023-06-01) across all methods (**Supplementary Tables 1**, **2**, **3**) to ensure a consistent and fair comparison across methods. We then used all the listed tools to generate and analyze five structural models per TCR-pMHC complex.

**Figure 1:**
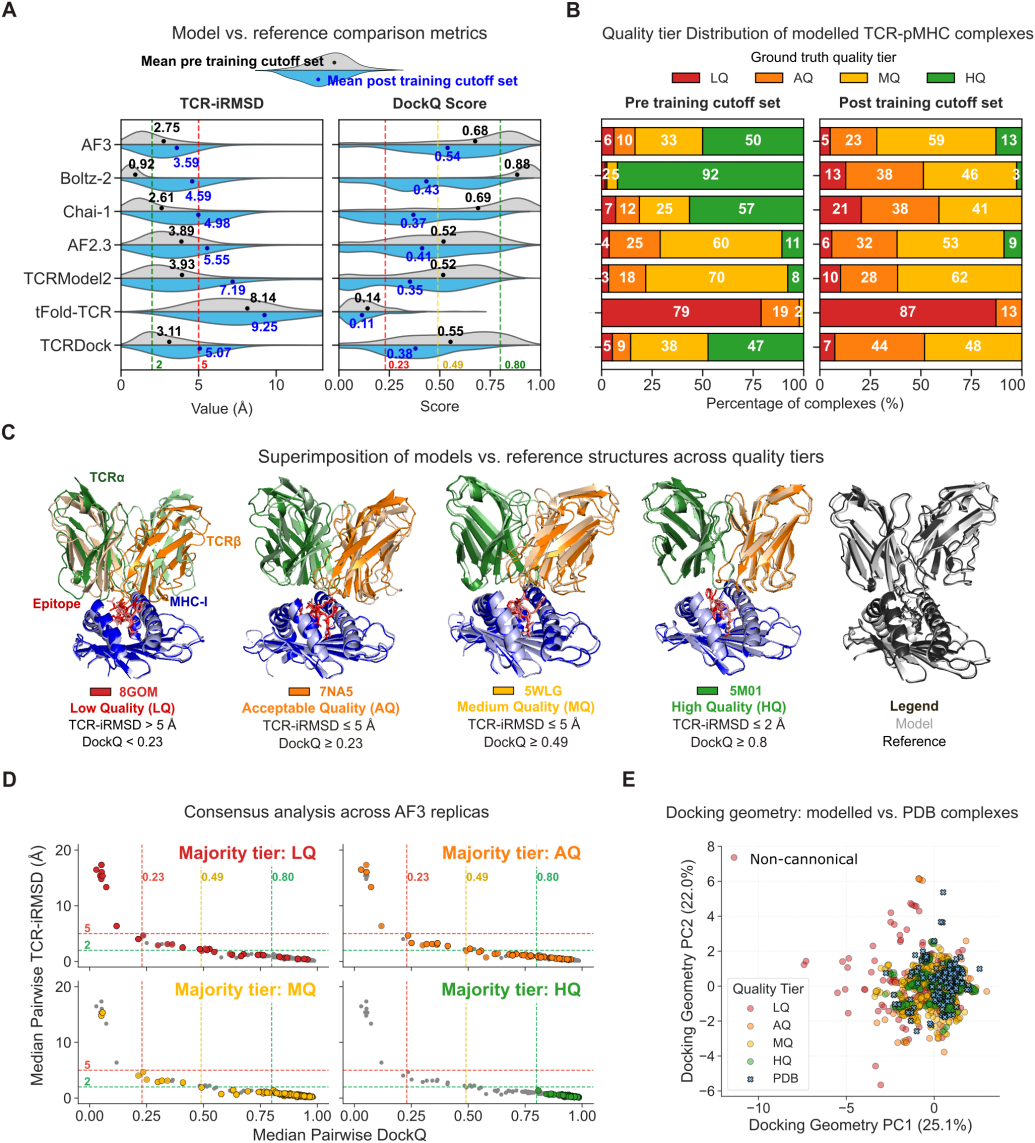
Benchmarking TCR-pMHC structure prediction tools against all reference structures in the PDB **(A)** Distributions of TCR α/β interface RMSD (TCR-iRMSD) and DockQ scores for model vs. experimental reference structure comparisons of TCR-pMHC class I complexes. Models were generated using state-of-the-art structure prediction tools, including general-purpose (AlphaFold3, Chai-1, Boltz-2, and AlphaFold-Multimer 2.3) and TCR-specific tools (TCRmodel2, tFold-TCR and TCRdock). Structures were divided based on deposition date in the PDB and the training date: before the training cutoff date (pre, gray) and after the training cutoff date (post, blue). For comparability, we applied the most recent cutoff (Boltz-2: 2023-06-01) across all methods. Numbers indicate the mean values of the metrics for each set. Dashed lines indicate established thresholds for structural quality classification. **(B)** Percentages of HQ (high quality), MQ (medium quality), AQ (acceptable quality), and LQ (low quality) assigned to the highest quality model per complex among the five generated AF3 replicas per TCR-pMHC complex. Left panel shows the pre-training cutoff set, while right panel shows the post-training cutoff set. **(C)** Criteria used to classify model quality into four tiers: HQ (high quality), MQ (medium quality), AQ (acceptable quality), and LQ (low quality), based on TCR-iRMSD and DockQ thresholds. Superimposed structures illustrate the reference structure (dark colors) versus the corresponding AlphaFold3 model (light colors) for representative PDB entries: LQ (8GOM, model 4), AQ (7NA5, model 2), MQ (5WLG, model 1), and HQ (5M01, model 0). **(D)** Scatter plots representing the median pairwise TCR-iRMSD and DockQ scores between AF3-modeled TCR-pMHC replicas. Each dot corresponds to the median values of both metrics between the comparisons of the five modelling replicas of a given TCR-pMHC complex, colored by the majority tier per complex. **(E)** Docking geometry landscape of PDB TCR-pMHC interfaces (blue crosses) and AlphaFold3-modeled TCR-pMHC interfaces (points colored by quality tier), visualized by projecting the 6-dimensional rigid-body transform space onto its top two principal components (PC1 and PC2).

We assessed model quality by comparing the predicted structures versus their corresponding experimental structures using multiple evaluation metrics (**Figure 1A**; **Supplementary Figure 1A**). Among these, as representative measures of TCR-pMHC model interface quality, we used the Cα interface RMSD of the TCR chains (TCR-iRMSD), which includes the six peptide-interacting CDR regions, and the DockQ (55) score of the TCR-pMHC interface. These two metrics were strongly but non-linearly correlated (**Supplementary Figure 1D**). Correlation analysis revealed a strong negative association (Spearman, ρ = -0.96), indicating that lower structural deviation of the TCR interface regions correspond to higher docking quality.

A marked performance gap emerged between pre- and post-cutoff structures across nearly all tools and metrics (**Figure 1A**; **Supplementary Figure 1A**). For TCR-iRMSD, Boltz-2 achieved the highest accuracy on pre-cutoff structures, with a mean of 0.92 Å, but performance decreased to 4.59 Å for post-cutoff structures. Chai-1 and AF3 showed similarly strong pre-cutoff performance (2.61 Å and 2.75 Å, respectively) yet declined on post-cutoff complexes (4.98 Å and 3.59 Å, respectively). DockQ scores followed a similar trend to TCR-iRMSD; Boltz-2 decreased from a high-quality DockQ mean of 0.88 (pre) to 0.43 (post), falling into the acceptable tier. AF2.3, TCRmodel2 and TCRdock showed comparable intermediate performance maintaining medium-quality predictions for pre-cutoff structures (TCR-iRMSD; 3.11-3.93, DockQ; 0.52-0.55) but declining moderately to acceptable quality (TCR-iRMSD; 5.07-5.55, DockQ; 0.35-0.41) for post-cutoff structures. In contrast, tFold-TCR remained in the low-quality tier, with DockQ scores of 0.14 (pre) and 0.11 (post) and TCR-iRMSD values exceeding 8 Å.

We next categorized the models into four ground truth quality tiers based on comparisons with the experimental references: high quality (HQ; TCR-iRMSD ≤ 2 Å and DockQ ≥ 0.8), medium quality (MQ; TCR-iRMSD ≤ 5 Å and DockQ ≥ 0.49), acceptable quality (AQ; TCR-iRMSD < 5 Å and DockQ ≥ 0.23), and low quality (LQ; TCR-iRMSD > 5 Å or DockQ < 0.23). Representative examples highlight the progressive deviations between predicted and experimental structures from higher to lower tiers. HQ models generally resemble the experimental structures closely, whereas LQ models sometimes exhibit extreme deviations, including complete swapping of the TCR α and β chains (**Figure 1C**).

We analyzed the highest-quality model among the five replicas generated per TCR-pMHC complex and observed trends mirroring those seen for the TCR-iRMSD and DockQ scores (**Figure 1B**): Boltz-2 performed best on pre-cutoff datasets, producing 92% high-quality (HQ) models. Chai-1, AF3 and TCRdock also showed strong performance on this pre-cutoff data, yielding 57%, 50% and 47% HQ models respectively, keeping over 90% of their predictions within the acceptable-to-high-quality (AQ-HQ) range. However, performance dropped across all methods on the post-cutoff datasets. This drop was particularly sharp for Boltz-2, Chai-1 and TCRdock (falling to 3% and 0% HQ models), indicating limited generalization capacity. In contrast, AF3 showed the strongest generalization beyond its training set. For post-cutoff complexes, it achieved 13% HQ and 59% medium-quality (MQ) models, with 95% of predictions falling within the acceptable-to-high-quality (AQ-HQ) range, outperforming the other methods on post-cutoff data. AF2.3, the next best-performing tool in post-cutoff complexes, also showed some generalization, maintaining 95% of predictions within the AQ-HQ range, though it produced roughly fewer HQ and MQ models compared to AF3. These trends were also consistently reproduced when using each method’s respective training cutoff sets, thereby expanding the evaluation dataset and reinforcing the same overall conclusions (**Supplementary Figures 1B, C**). Importantly, sequence and structural similarity analyses, although showing some correlation with more similar cases being more accurately modeled, reveal substantial overlap in similarity distributions across quality tiers. Some LQ cases exhibit similarity levels comparable to MQ, AQ, and HQ cases. The same pattern is also observed in the opposite direction, with some higher-quality cases showing similarity levels typical of lower-quality groups. This indicates that performance is not necessarily driven by proximity to training data but instead reflects generalization ability (**Supplementary Figures 2**, **3**).

Given its better generalization performance, we further evaluated the structural consistency of AF3 predictions by studying the agreement amongst the 5 model replicas generated for each TCR-pMHC complex. We expected that complexes showing higher agreement across replicas would correspond to more reliable predictions. To this end, we computed the median pairwise TCR-iRMSD and DockQ scores across replicas and compared them to the ground-truth quality tiers defined relative to the experimental references (**Figure 1D**). Consensus across model replicas strongly correlated with model accuracy. HQ and MQ models exhibited low pairwise median TCR-iRMSD and high pairwise median DockQ values, reflecting robust structural convergence, whereas AQ and LQ models, showed greater dispersion. This relationship was supported by strong Spearman correlations between median pairwise and model versus experimental reference metrics (ρ = 0.76 for TCR-iRMSD; ρ = 0.79 for DockQ) (**Supplementary Figure 1E**).

Finally, we analyzed the docking geometries of AF3-predicted structural models using six rigid-body orientational parameters describing the relative orientation between the pMHC and the TCR subunits. These parameters were computed with TCRdock software and visualized using principal component analysis (PCA) (**Figure 1E**). HQ models closely reproduced the TCR-pMHC binding modes observed in experimental references, whereas lower-quality models showed progressively larger deviations from canonical binding geometries, with the lowest-quality models sometimes adopting non-canonical docking orientations (**Supplementary Figure 1F**).

### 2.2 Combining modelling confidence metrics enhances structural quality classification for TCR-pMHC class I models

To enable large-scale evaluation of synthetic structural data from annotated TCR-pMHC sequences, we aimed to develop a reference-free quality tier classification system exclusively based on model confidence metrics. To this end, we evaluated multiple metrics that can be directly extracted from predicted structures without requiring experimental references. These span several levels of structural assessment: structural confidence measured using predicted local distance difference test scores (pLDDT) for the full complex and for subsets focusing on CDRα/β regions; protein-protein interface confidence metrics such as the interface predicted TM-score (ipTM) (35) and a TCR-pMHC-specific ipTM (29), the average interface predicted aligned error (iPAE) scaled to yield a dimensionless confidence score (39), the average interface predicted distance error (iPDE) (36) and the interaction prediction score from aligned errors (ipSAE) (37). Additionally, we evaluated the predicted DockQ scores v1 (38) and v2 (39), computed specifically for the TCR-pMHC interface.

**Figure 2:**
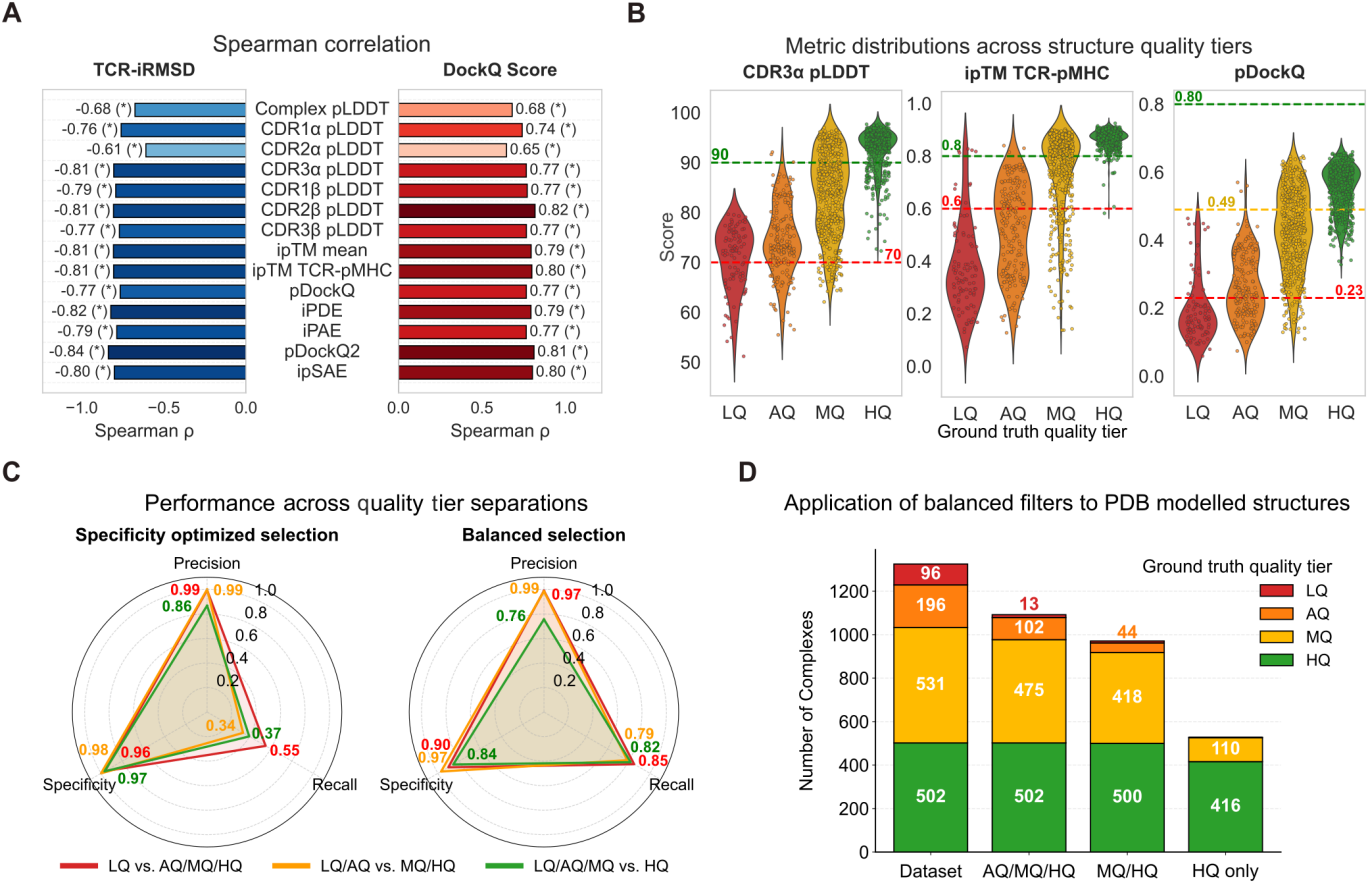
Evaluation of the structural quality of AlphaFold3 TCR-pMHC class I predictions using modeling confidence metrics. **(A)** Spearman correlation coefficients between model confidence metrics and model vs. experimental reference comparison metrics. Model confidence metrics include full complex, CDR1, CDR2, and CDR3α, as well as CDR1, CDR2, and CDR3β pLDDTs, mean ipTM, TCR-pMHC-specific ipTM, ipSAE, and predicted DockQ scores (v1 and v2). Model vs. experimental reference comparison metrics include TCR-iRMSD (blue) and DockQ scores (red) (* p-value < 0.001). **(B)** Distribution of AF3 model confidence metrics including CDR3β pLDDT, TCR-pMHC-specific ipTM, and predicted DockQ scores. Distributions are stratified by quality tiers: Low Quality (LQ), Acceptable Quality (AQ), Medium Quality (MQ) and High Quality (HQ). Red dashed lines indicate acceptability thresholds; yellow lines denote medium-quality thresholds; and green lines indicate high-quality thresholds. **(C)** Radar plots showing the optimal combinations of metrics identified to progressively discriminate between adjacent quality tiers with maximum precision or Specificity-Recall balance. **(D)** Sequential effect on model quality distribution after applying the balanced metric-based filters to the AF3-modeled PDB TCR-pMHC Class I dataset. Models are classified using boolean operators (AND/OR) based on model confidence metrics and their established thresholds through a three-stage process.

All model confidence metrics significantly correlated with model versus experimental reference metrics. Specifically, TCR-iRMSD and DockQ scores, exhibited the strongest correlations with model confidence metrics, with Spearman coefficients (ρ) ranging from -0.61 to -0.84 and from 0.65 to 0.82 respectively (**Figure 2A**; **Supplementary Figure 4A**). Interface-related variables such as pDockQ2 and iPDE stood out as highly reliable predictors of model accuracy, showing Spearman ρ values of 0.84 and 0.82 with TCR-iRMSD, and 0.81 and 0.79 with DockQ, respectively. These trends were further reflected in the distribution of confidence metrics across ground truth quality tiers (LQ, AQ, MQ, and HQ). The distribution of confidence metrics increased progressively with higher structural accuracy (**Figure 2B**; **Supplementary Figure 4B**). Across metrics, HQ models formed compact clusters at the upper end of the score distributions, particularly for pLDDT-based metrics such as CDR3β and the TCR-pMHC ipTM. Despite these correlations, a moderate degree of distribution overlap persisted across tiers for most metrics, highlighting the limited discriminative power of individual metrics. For example, CDR3β pLDDT and TCR-pMHC ipTM correctly identified over 90% of HQ models but carried the inclusion of 45% of the MQ models when using the high-quality pLDDT threshold of 90% (**Figure 2B**). Leakage was even greater across lower-quality tiers (LQ, AQ, MQ). Misclassification was most notable for pDockQ, where over 50% of MQ models fell below the LQ threshold (0.23), and values never exceeded the HQ threshold (0.80), even for the most accurate models.

Observing that individual confidence metrics rely on distinct cutoffs to classify quality tiers and exhibit systematic biases, we next evaluated whether combining these metrics could improve tier discrimination while prioritizing higher-quality TCR-pMHC models. We tested exhaustive combinations of confidence metrics and thresholds using a five-fold cross-validation framework, evaluating a total of 265,720 integrative filters with varying discriminative capacities (**Supplementary Figure 4C**). Given the class imbalance in the dataset, with 7% LQ models (n=96), 15% AQ models (n=196), 40% MQ models (n=531), and 38% HQ models (n=502), we focused on optimizing precision and specificity to capture performance on both the positive and negative classes. This ensured that higher-quality models were reliably identified while minimizing the inclusion of lower-quality structures. Filters optimized for maximum specificity effectively removed lower-quality models (**Figure 2C**), achieving high precision (0.99, 0.99, 0.86) and specificity (0.96, 0.98, 0.97) for LQ vs. AQ/MQ/HQ, LQ/AQ vs. MQ/HQ, and LQ/AQ/MQ vs. HQ, respectively. However, recall remained relatively low (0.55, 0.34, 0.37). In contrast, balanced filters, optimized using the harmonic mean of specificity and recall, provided more consistent performance across tiers. Precision remained high (0.97, 0.95, 0.76), while recall improved substantially (0.85, 0.79, 0.82), and specificity decreased modestly (0.90, 0.97, 0.84) for the same comparisons (**Figure 2C**).

Building on these results, we applied the balanced filters (**Table 1**) to the full dataset to evaluate their practical impact on tier stratification (**Figure 2D**). Model quality was assigned according to whether each model passed the selected criteria. The first filter (Filter LQ) removed ~86% of LQ models, demonstrating high discriminatory power against low-quality structures. However, it also excluded a substantial fraction of higher-quality models (~50% of AQ and ~10% of MQ), reflecting the precision-recall trade-off observed during cross-validation. The second filter (Filter AQ) eliminated 4 additional LQ models and ~60% of the remaining AQ models, while also removing ~12% of the remaining MQ models and 2 HQ models, again reflecting lower recalls. Finally, the third filter (Filter MQ) removed approximately 74% of the remaining MQ models while excluding ~17% of HQ models, thereby enriching the retained subset for the highest-quality structures.

**Table 1:** Metric combination filters for TCR-pMHC model quality tier assignment.

Classification level	Logic and hierarchical metric thresholds
LQ vs. AQ/MQ/HQ	pLDDT CDR1α > 70 AND pLDDT CDR3α > 70 AND pLDDT CDR1β > 70 AND pLDDT CDR2β > 70 AND pLDDT CDR3β > 70 OR ipSAE > 0.60 OR ipTM (TCR-pMHC) > 0.60 AND pDockQ > 0.23 OR pDockQ2 > 0.23
LQ/AQ vs. MQ/HQ	pLDDT Complex > 70 OR pLDDT CDR1α > 70 AND pLDDT CDR2α > 70 OR pLDDT CDR3α > 70 AND pLDDT CDR1β > 70 OR pLDDT CDR2β > 70 OR pLDDT CDR3β > 70 OR ipSAE > 0.60 OR ipTM (TCR-pMHC) > 0.60 OR pDockQ2 > 0.49
LQ/AQ/MQ vs. HQ	pLDDT Complex > 90 OR pLDDT CDR1α > 90 AND pLDDT CDR2α > 90 OR pLDDT CDR3α > 90 AND pLDDT CDR2β > 90 AND pLDDT CDR3β > 90 AND ipTM > 0.80

Summary table of the filtering logic used to categorize models into four quality tiers selected by optimizing the specificity-recall balance. Models are classified using boolean operators (AND/OR) based on model confidence metrics and their established thresholds.

Overall, combining multiple model confidence metrics can improve the discrimination of TCR-pMHC structural quality tiers, particularly in identifying high- and low-quality models. However, the approach remains dependent on manually defined thresholds and complex combinations of metrics, which can limit recall and overall generalizability. These limitations suggest that further refinement or alternative strategies may be needed to achieve more robust, threshold-independent classification.

### 2.3 Random forest provides a probabilistic and interpretable structural quality classification for TCR-pMHC models

To overcome the limitations of single-metric thresholding and systematic biases observed in individual confidence metrics, we integrated all evaluated metrics into a random forest (RF) classifier. While we also tested more complex models such as XGBoost and simpler ones such as logistic regression (**Supplementary Figure 5**), RF achieved comparable performance without added complexity. The evaluated confidence metrics are particularly well-suited for tree-based models, as they can capture non-linear interactions, handle differing scales effectively, and accommodate the high correlations among metrics. Furthermore, RF provides a lightweight and easily tunable framework while allowing for interpretability using SHAP values, which was essential for understanding the contributions of individual confidence metrics.

Based on these considerations, we trained a multiclass classifier to distinguish between our four structural quality tiers (LQ, AQ, MQ, HQ) derived from model versus experimental reference comparisons (**Figure 1A**). The RF classifier integrates global and local confidence descriptors described previously as input features (full-complex pLDDT, CDRα/β-pLDDTs, ipTM, TCR-pMHC ipTM, iPAE, iPDE, ipSAE, and pDockQ scores v1-2). By using a probabilistic framework, the classifier enables reference-free assignment of models to quality tiers, improving both discrimination and interpretability compared with individual or combined confidence metrics using fixed thresholds.

**Figure 3:**
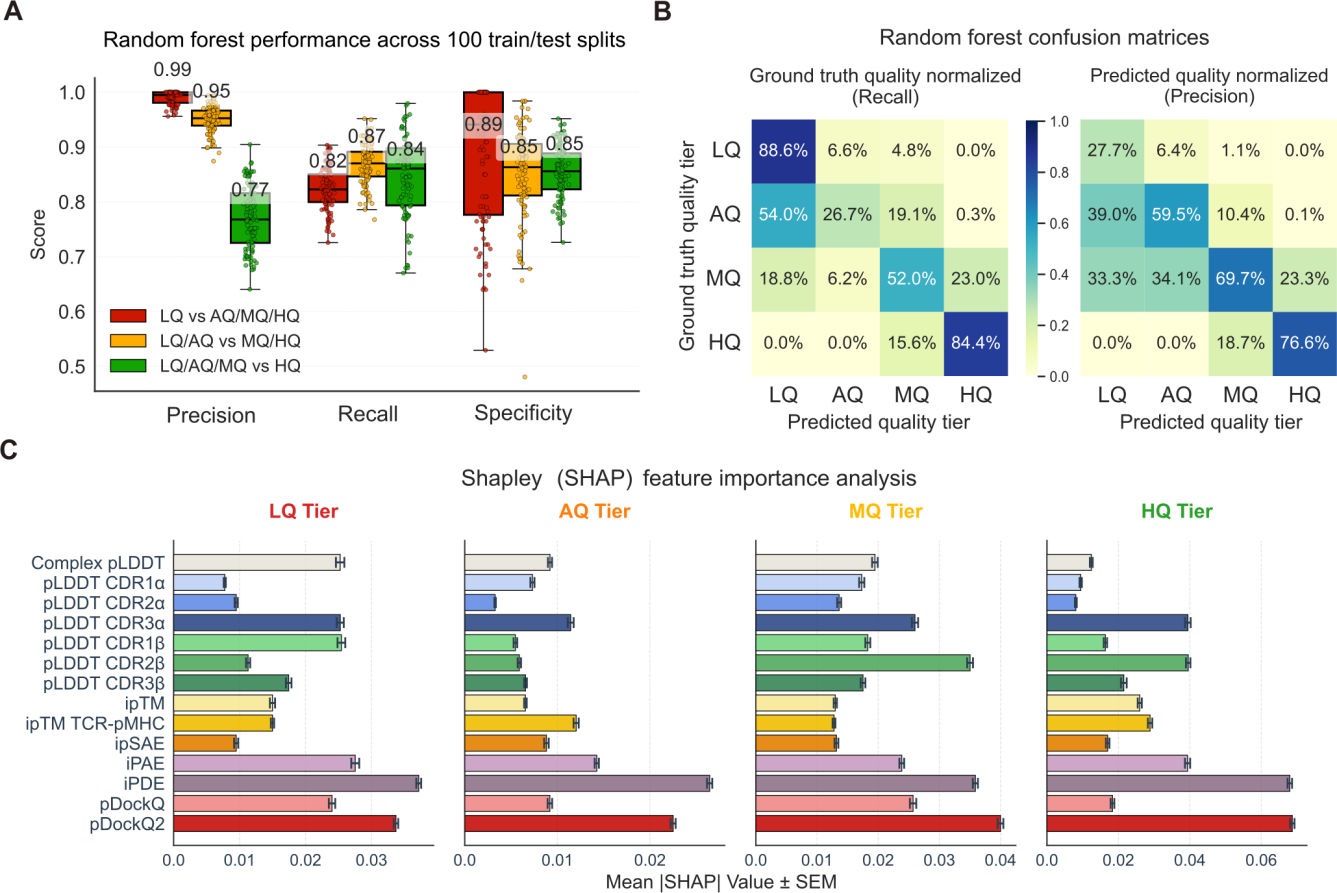
Random forest classifier for structural quality tier assignment: performance and feature contribution analysis. **(A)** Performance metrics (Precision, Recall, and Specificity) of the random forest (RF) classification across three different classification setups: Low Quality vs. rest (LQ vs rest; red), Low/Acceptable Quality vs. Medium/High Quality (LQ/AQ vs MQ/HQ; orange), and Low/Acceptable/Medium Quality vs. High Quality (LQ/AQ/MQ vs HQ; green). The classifier was trained to distinguish quality tiers (LQ, AQ, MQ, and HQ) derived from experimental-versus-model structure comparisons using modelling confidence metrics. Boxplots and individual data points represent test set evaluations across 100 independent random initialization seeds, with median values indicated above each plot. **(B)** Confusion matrices showing multiclass prediction performance across the four structural quality tiers (LQ, AQ, MQ, HQ). Left matrix is normalized by the ground truth quality tier focusing on recall. Right matrix is normalized by predicted quality tier focusing on precision. **(C)** Shapley feature importance (SHAP) values for structural model confidence metrics, including pLDDT (full complex and CDRs of the α and β chains), mean ipTM, TCR-pMHC-specific ipTM, ipSAE, averaged scaled iPAE, averaged iPDE, pDockQ, and pDockQ2, reported separately for each quality tier to illustrate tier-specific feature contributions to RF classification.

Performance was evaluated across 100 independent train/test splits to assess consistency over different initialization points (**Figure 3A**). The RF classifier achieved a high and stable performance across splits, indicating limited sensitivity to sampling variability. When discriminating LQ models from higher tiers (LQ vs. AQ/MQ/HQ), precision reached 0.99 and recall 0.82, with a specificity of 0.89. For the intermediate grouping (LQ/AQ vs MQ/HQ), performance remained balanced, with precision of 0.95, recall of 0.87, and specificity of 0.85. Discriminating HQ models from all other tiers (LQ/AQ/MQ vs HQ) proved more complex, displaying lower precision (0.77) but maintaining recall of 0.84 and specificity (0.85). Overall discrimination was comparable to that achieved by metric-based filtering (**Figure 3C**) indicating reliable identification of higher-quality structures while minimizing false positive assignments. However, the RF classifier yielded consistent performance across splits and enabled probabilistic multiclass predictions, allowing continuous confidence estimates rather than binary inclusion/exclusion decisions.

The confusion matrix (**Figure 3B**) further illustrates how the classification behavior of our RF classifier varies across quality tiers. LQ and HQ tiers were correctly identified in 88.6% and 84.4% of cases, respectively, confirming clear separation at the extremes of the quality spectrum. Misclassification predominantly occurred between adjacent tiers. Among predicted LQ structures, 39.0% were AQ and 33.3% MQ, reflecting a deliberate trade-off: recall was limited to improve precision and reduce false positives. Importantly, misassignments over non-contiguous tiers were not observed: no HQ structures were classified as LQ or AQ, and no LQ structures were predicted as HQ (0.0%), preserving the global quality hierarchy. Many errors correspond to upward shifts between contiguous tiers, MQ predicted as AQ (34.1%), or HQ predicted as MQ (18.7%). Nevertheless, these shifts do not compromise the primary objective of separating lower-quality from higher-quality model. True MQ models were predicted as MQ or HQ in 75% of cases, whereas LQ models were predicted as AQ, MQ, or HQ in only 11.4% of cases.

Lastly, to characterize feature contributions within our RF classifier, we computed SHAP (56) (SHapley Additive exPlanations) values for each class (**Figure 3C**). Across quality tiers, interface-focused metrics such as pDockQ2 and iPDE consistently contributed strongly to tier prediction. This indicates that docking confidence and interface geometry are central determinants of structural quality stratification. This observation aligns with the strong Spearman correlations observed between these metrics and TCR-iRMSD and DockQ scores (**Figure 2A**). In the LQ tier, additional contributions were observed from full-complex pLDDT and specific CDR loop confidence values, particularly CDR3α and CDR1β, reflecting the widespread reduction in local and global confidence of these models. In the AQ tier, feature contributions were more evenly distributed across metrics, consistent with intermediate structural reliability. In the MQ and HQ tiers, interface metrics including iPAE and CDR3α/β pLDDT values gained relative importance, highlighting the increasing contribution of precise loop modeling and interface refinement at higher quality levels.

### 2.4 Quality tier stratification enriches for experimentally validating TCR-pMHC interactions in TCRvdb

**Figure 4:**
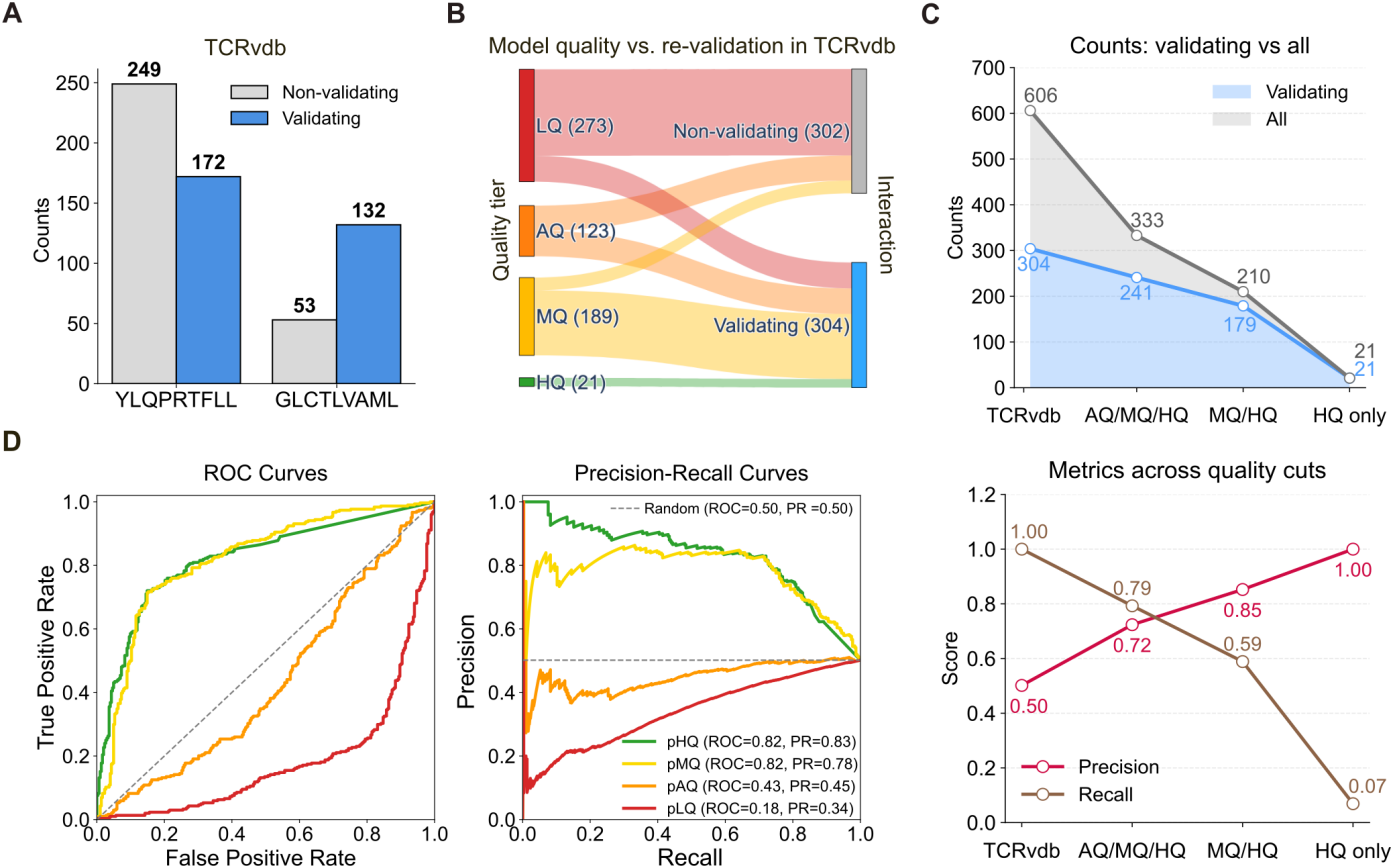
Quality tier stratification enriches for experimentally validating TCR-pMHC interactions in TCRvdb. **(A)** Counts of validating (blue) versus non-validating (gray) TCR-pMHC complexes for the epitopes YLQPRTFLL and GLCTLVAML. **(B)** Alluvial plot showing the distribution of quality tiers Low Quality (LQ), Acceptable Quality (AQ), Medium Quality (MQ) and High Quality (HQ), among validating and non-validating complexes **(C)** Counts of validating and non-validating complexes after progressive removal of lower-quality tiers, with recall and precision reported at each filtering step. **(D)** ROC and PR curves of the random forest-predicted probabilities for each quality tier (probability of being low quality pLQ, acceptable quality pAQ, medium quality pMQ and high quality pHQ). Random expectation is indicated with a dashed line.

The TCR validated database (TCRvdb) (50) provides a reevaluation of two frequent immunodominant epitopes annotated at VDJdb (49), revealing that a substantial amount of annotated interactions (~50%) could not be experimentally revalidated. These epitopes are GLCTLVAML (Epstein-Barr virus) and YLQPRTFLL (SARS-CoV-2), each paired with multiple TCRs and restricted to HLA-A*02:01. We leveraged this dataset from Messemaker et al., which was generated by testing complexes in a harmonized T-cell validation system (57). This setup allowed the identification of TCR-pMHC complexes with experimentally validating and non-validating interactions. Specifically, 249 YLQ complexes were reevaluated as non-validating and 172 as validating, while 53 GLC complexes were non-validating and 132 validating (**Figure 4A**; **Supplementary Tables 1**, **2**). Building on this dataset, we investigated whether predicted structural quality is associated with experimental validation status. To this end, we modeled these TCR-pMHC triads using AF3 and assessed whether validating interactions were enriched among higher-quality models classified using our RF-based quality tiers. For downstream analyses, we selected a single representative structure per TCR-pMHC complex amongst the five AF3-generated replicas, prioritizing the highest RF-assigned quality tier. When multiple replicas shared the same tier, we selected the structural model with the highest classifier probability score.

Our analysis revealed that our structural quality tier approach is associated with TCR-pMHC interactions that were revalidated experimentally using state-of-the-art T-cell screenings (50) (**Figure 4B**). We observed that LQ models are predominantly associated with non-validating interactions, whereas higher-quality tiers exhibit progressive enrichment for validating complexes. This culminates in the HQ tier, which consists exclusively of positive TCR-pMHC interactions. This association is quantitatively supported by a Chi-square (χ²) test and Odds Ratio (OR) analysis (**Supplementary Figure 6A**), revealing a pronounced statistical divergence: LQ models were significantly depleted of validating interactions (OR = 0.12) and enriched for non-validating ones (OR = 8.65), while medium quality (MQ) models were enriched for validating interactions (OR = 9.33) and depleted of non-validating ones (OR = 0.11). Between these extremes, the Acceptable-Quality (AQ) tier functions as a non-significant (ns) transition zone, hovering near the random expectation (OR ≈ 1.00), indicating roughly equal numbers of validating and non-validating interactions.

Subsequently, we evaluated the precision and recall values of progressively excluding lower-quality tiers to determine whether retaining only higher-confidence structural models effectively purges non-validating interactions from the dataset. The total TCRvdb dataset, initially comprising 606 complexes with approximately 50% of validating and 50% of non-validating TCR-pMHC complexes (baseline precision of 0.50 and full recall 1.00), underwent significant refinement in higher quality tiers (**Figure 4C**). Excluding LQ models (AQ/MQ/HQ cut) reduced the total count from 606 to 333 complexes, removing most non-validating ones (210 out of 302), increasing precision to 0.72 while maintaining a recall of 0.79 (241 positives out of 333 complexes). Further stringency (MQ/HQ cut) deepened this enrichment, raising precision to 0.85, though at the cost of recall dropping to 0.59 (179 positives out of 210 complexes). Notably, at the highest threshold (HQ only), the model achieved absolute precision (1.00), identifying 21 complexes that were all experimentally validating.

In parallel, we assessed the continuous discriminative power of our RF classifier for distinguishing validating from non-validating TCR-pMHC interactions. Unlike the discrete tier-based filters, which classify models into LQ, AQ, MQ, and HQ, this analysis leverages the full range of predicted probabilities assigned by the RF. We analyzed these probabilities for each quality tier using ROC and PR curves and quantified pairwise differences with the DeLong test and Bonferroni correction (**Figure 4D**; **Supplementary Figure 6B**). Low-Quality (LQ) probabilities showed minimal discriminative capacity, with a ROC AUC of 0.18 and PR AUC near 0.34, reflecting their strong association with non-validating interactions. Medium-Quality (MQ) and High-Quality (HQ) predictors exhibited consistent performance, with no significant differences (ROC AUCs of 0.82 and PR AUCs of 0.78 and 0.83 respectively, p_adj_ = 1.00, indicating that an MQ threshold is sufficient to enrich for validating complexes. Acceptable-Quality (AQ) probabilities occupied an intermediate position (ROC AUC = 0.43, PR AUC = 0.45), consistent with their non-significant Odds Ratios.

### 2.5 IMMREP23 dataset shows enrichment of positive interactions amongst higher-quality TCR-pMHC structural models

We next investigated whether modelled structural quality could distinguish true TCR-pMHC positive triads from synthetic negatives generated by swapping TCRs across unrelated pMHCs. To this end we leveraged the IMMREP23 (46) TCR-pMHC dataset from Kaggle, which comprises 3,484 TCR-pMHC class I complexes spanning 20 epitopes. This dataset reflects a broader biological diversity than TCRvdb and includes epitopes both present (7/20) and absent (13/20) in experimentally solved TCR-pMHC structures. In addition, the dataset is inherently imbalanced (~1:5 positive-to-negative ratio at epitope level), thus representing a more realistic large-scale TCR screening scenario. We modeled these 3,484 TCR-pMHC structures using AF3 and classified them into quality tiers (LQ, AQ, MQ, HQ) using our random forest (RF) classifier (**Supplementary Tables 1**, **2**). For downstream analyses, we selected a single representative structure per TCR-pMHC complex amongst the five AF3-generated replicas, prioritizing the highest RF-assigned quality tier. When multiple replicas shared the same tier, we selected the structural model with the highest classifier probability score.

**Figure 5:**
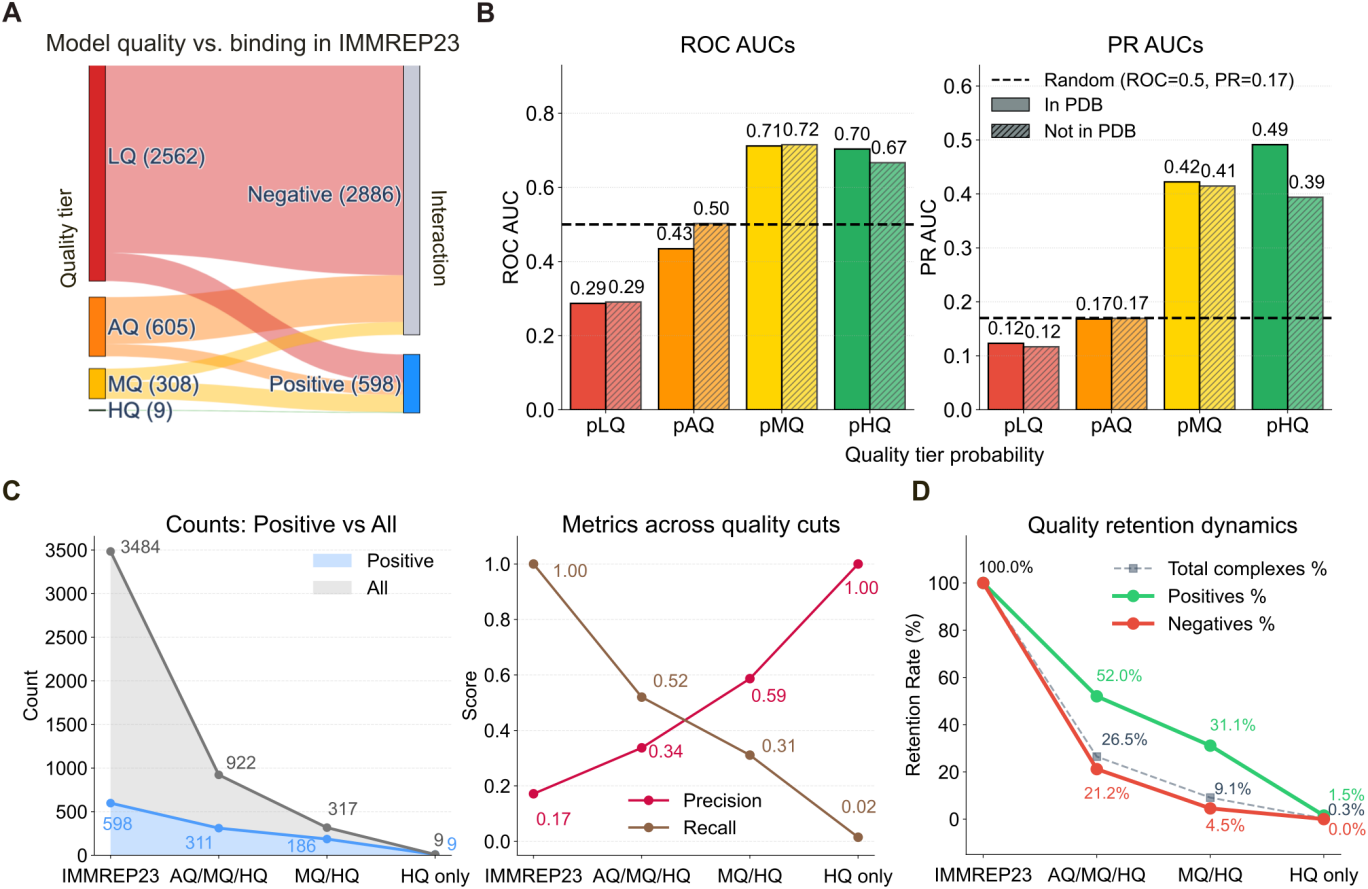
Higher-quality structural models enrich for positive TCR-pMHC interactions in the IMMREP23 dataset. **(A)** Alluvial plot showing the distribution of structural quality tiers; Low Quality (LQ), Acceptable Quality (AQ), Medium Quality (MQ) and High Quality (HQ), among positive and synthetic negative TCR-pMHC triads. **(B)** ROC and PR AUCs of the probabilities assigned by the Random Forest model for each quality tier (LQ: red, AQ: orange, MQ: yellow, HQ: green), split by whether the epitopes are present in experimentally solved PDB complexes (Present in PDB: solid bars) or absent (Not in PDB: dashed bars). **(C)** Counts of positive and negative TCR-pMHC complexes after progressive removal of lower-quality tiers, with recall and precision indicated for each step. **(D)** Changes in the proportions of total complexes (gray), positives (green), and negatives (red) as progressively lower-quality tiers are removed from the IMMREP23 dataset.

The distribution across tiers was markedly skewed, with most structures classified as LQ (2,562), followed by AQ (605), MQ (308), and only 9 HQ models. Consistent with the stratification pattern observed before in TCRvdb, positive complexes were progressively enriched in higher-quality tiers, whereas LQ models were predominantly associated with negatives (**Figure 5A**). This enrichment was quantitatively supported by Odds Ratio analysis (χ² test) (**Supplementary Figure 7A**). LQ models contain significantly fewer true binders (OR = 0.25) and are strongly enriched in non-binders (OR = 4.03). AQ showed a modest but statistically significant enrichment for positives (OR = 1.33) and a corresponding depletion in negatives (OR = 0.75). In contrast, MQ models displayed a strong positive association (OR = 8.82) and a marked depletion of negatives (OR = 0.11).

Again, we analyzed the RF probabilistic performance computing ROC and PR curves (**Figure 5B**). MQ and HQ probabilities provided strong discriminative performance in the global analysis (ROC AUC = 0.67-0.72; PR AUC = 0.39-0.49), substantially outperforming LQ (ROC AUC = 0.29; PR AUC = 0.12) and AQ predictors (ROC AUC = 0.43-0.50; PR AUC = 0.17). Performance remained consistent across epitopes present (7/20) or absent (13/20) in experimentally solved TCR-pMHC structures. MQ performance was largely stable between groups, whereas HQ showed a moderate decrease in unseen epitopes (ROC AUC from 0.70 to 0.67; PR AUC from 0.49 to 0.39), indicating partial dependence on similarity to known complexes but overall generalizability beyond the training distribution. Per-epitope analyses mirrored these global trends (**Supplementary Figures 7B, C**). Mean ROC AUC values were 0.66 and 0.64 for HQ and 0.69 and 0.68 for MQ for epitopes present and absent from the PDB structures, respectively, with greater variance among epitopes absent from the PDB. Mean PR AUC values were 0.41 (MQ) and 0.44 (HQ) for epitopes represented in structures, decreasing modestly to 0.39 and 0.37 respectively, for epitopes absent from PDB TCR-pMHC structures.

To further investigate enrichment, we analyzed the dataset across discrete predicted quality tiers, progressively excluding lower-quality models. Applying increasingly stringent quality filters produced substantial gains in precision at the expense of recall (**Figure 5B**). The full dataset exhibited low precision (0.17), consistent with class imbalance (598 positives of 3484 complexes). Excluding LQ models (AQ/MQ/HQ threshold) nearly doubled precision to 0.34 while retaining 0.52 recall (311 positives out of 922 complexes). Restricting to MQ/HQ further increased precision to 0.59, with recall declining to 0.31 (186 positives out of 317 complexes). At the highest stringency (HQ only), perfect precision (1.00) was achieved, albeit with minimal recall (0.02) with only 9 positive complexes. The filtering framework demonstrates its ability to achieve enrichment by effectively de-noising the TCR-pMHC interaction space. (**Figure 5D**). By systematically excluding LQ models, the total number of complexes is reduced to 26.5% of the initial library while retaining 52% of all positive interactions. As stringency increases to the MQ/HQ levels, the retention of synthetic negatives drops sharply to just 4.5%, while maintaining 31.1% of the positives in a dataset reduced by 90%. Ultimately, at the HQ tier, no negative triads remain, whilst 1.5% of positive complexes are enriched in 0.3% of the dataset, highlighting the specificity high-quality structural models.

## 3 Discussion

The structural prediction of TCR-pMHC interactions remains a major challenge in computational immunology due to the vast diversity of the TCR repertoire and the subtle biophysical cues that govern pMHC recognition (4, 8). Here, we present a scalable and interpretable framework for assessing the quality of TCR-pMHC class I structural models generated using state-of-the-art protein structure predictors, with a particular focus on AlphaFold3 (25) (AF3).

Our benchmark demonstrates that AF3 reliably predicts TCR-pMHC complexes not included in its training set, performing better than other general-purpose and TCR-specific methods (**Figure 1A**). Diffusion-based models, such as Boltz-2 (27) and Chai-1 (26), achieve high accuracy on structures included in their training set but their performance drops dramatically on novel complexes. tFold-TCR (30) consistently yields low-quality predictions, highlighting that MSA-free methods remain substantially less accurate than MSA-dependent approaches (33). In contrast, AF3 (25) maintains over 90% acceptable-quality predictions, of which ~13% are high-quality, in complexes absent from its training set (**Figure 1C**). Moreover, consensus across independent model replicas obtained from the same TCR-pMHC sequences, correlates positively with overall structural quality (**Figure 1D**), indicating that agreement between replicas is a useful proxy for structural prediction reliability (58). At the same time, AF3 models classified as lower-quality when compared to the experimental reference structure show moderate deviations in docking geometries and less accurately recapitulate CDR-mediated interactions observed in experimental TCR-pMHC structures (**Figure 1E**), potentially reflecting a bias toward docking modes present in the AF3 training set.

In our benchmark, AF3 emerged as the best-performing protein modelling tool; accordingly, we focused subsequent analyses on AF3-derived structural models. To systematically assess TCR-pMHC structural models, we used confidence metrics intrinsic to the modeling process including pLDDT, ipTM, iPAE, iPDE and pDockQ v1–2 scores to stratify complexes into four ground truth quality tiers based on agreement with experimental reference structures: low (LQ), acceptable (AQ), medium (MQ), and high (HQ). However, individual confidence metrics are insufficient to discriminate model quality due to overlapping distribution across adjacent tiers (**Figure 2B**).

Combining multiple metrics can improve tier classification (**Figure 2C**; **Table 1**); however, this approach typically relies on manually defined thresholds and exhaustive testing of many metric combinations, resulting in highly complex filters. Even with cross-validation, such reliance on intricate metric combinations can lead to overfitting to the calibration dataset, capturing patterns that may not generalize to new TCR-pMHC models. Moreover, each filter produces binary inclusion/exclusion decisions that cannot be fine-tuned, limiting flexibility for a more fine-grained assessment of structural quality. Together, these factors make threshold-based approaches less suitable for high-throughput or large-scale structural evaluations, highlighting the need for more continuous, and threshold-independent classification strategies. To alleviate this, we integrated these confidence metrics (full-complex pLDDT, CDRα- and CDRβ-specific pLDDTs, mean ipTM, TCR-pMHC-specific ipTM, iPAE, iPDE, ipSAE, and pDockQ scores v1-2) using a random forest classifier. This approach provided a simple yet powerful probabilistic score to discriminate quality tiers, enabling a fine-grained and quantitative assessment of structural modelling. This method allows flexible adjustment of decision thresholds to prioritize recall or precision for specific applications, is computationally efficient, and can handle correlated features, leading to more stable predictions and feature-based interpretations. Interface docking confidence scores, particularly pDockQ2 (39) and iPDE (36), are among the most correlated features with model versus experimental reference comparison metrics (**Figure 2A**) and consistently drive random forest predictions, as demonstrated by SHAP (56) analysis (**Figure 3C**). This highlights the central role of interface geometry in defining functional TCR-pMHC recognition. Furthermore, our random forest predictor is readily retrainable to incorporate additional structural models and can seamlessly integrate new quality metrics, enhancing adaptability and long-term utility.

Importantly, our model quality tiers are not only reflective of structural confidence, but are able to enrich for validated TCR-pMHC interactions. As a use-case, we studied a recent reassessment of TCR-pMHC triads in VDJdb (49), which revealed that ~50% of the interactions annotated for two immunodominant epitopes do not revalidate (50) using state-of -the-art T-cell screenings. In this context, our approach helps discriminate between validating and non-validating interactions (**Figure 4**): LQ models are strongly associated with non-validating triads, whereas MQ and HQ models are progressively enriched for validating ones. Similarly, in the IMMREP23 dataset (46), comparison of positive TCR-pMHC triads against synthetic negatives generated by swapping TCRs across unrelated pMHCs revealed an enrichment of positive interactions among higher-quality tiers (**Figure 5**). Although a similar enrichment trend to that observed in TCRvdb was present in IMMREP23, the magnitude of the association was weaker, consistent with the greater heterogeneity and class imbalance of the IMMREP23 dataset. Notably, MQ and HQ predictions retained discriminatory capacity for epitopes not represented in any of the TCR-pMHC complexes in the PDB, supporting the potential of structural quality stratification to generalize to unseen epitopes.

Our quality-tiered approach extends structural model quality assessment to settings in which experimentally resolved TCR-pMHC structures are unavailable, enabling the evaluation of models generated from sequence-derived databases such as VDJdb (49), IEDB (59), and McPAS (60). This, in turn, supports downstream applications, such as structure-based TCR-pMHC specificity prediction (12–15). Filtering or down-weighting low-quality structural models in training datasets may reduce noise and help predictive models learn from more reliable structural information.

Beyond model evaluation, our work motivates an end-to-end strategy for next-generation TCR-pMHC specificity prediction. Such methods may benefit from a modular architecture that separates structure generation, uncertainty estimation, quality assessment, and functional specificity prediction. In such frameworks, protein modeling tools such as AF3 could serve as the primary engine for generating candidate TCR-pMHC structural ensembles. Multiple independent replicas generated from different random seeds could then be compared to estimate conformational consistency and prediction uncertainty. An interface-focused quality assessment, such as the RF classifier introduced here, could stratify models into quality tiers and prioritize structurally plausible candidates before more computationally demanding downstream analyses. These downstream modules could include physics-based rescoring, molecular dynamics-based refinement, and ensemble-based scoring, ultimately leading to functional TCR-pMHC pairing or specificity prediction (10, 11, 14, 61–63). By reducing the number of low-confidence structures, reference-free quality assessment could improve the efficiency, interpretability, and scalability of downstream TCR-pMHC structural analyses. Thus, our framework is not only an AF3-centered structural model quality assessment strategy but also provides a conceptual basis for integrating structural modeling and functional prediction in next-generation TCR-pMHC specificity prediction, ultimately reducing the need for extensive experimental screening (64, 65).

Complementarily to evaluating naturally occurring TCR-pMHC interactions, model confidence metrics are commonly applied in computational protein design, including TCR (66) and antibody (67) engineering. In these fields, designs are typically filtered based on structural and interface metrics. However, experimental success rates often remain low, with only a small fraction of prioritized candidates, frequently below 5-10%, yielding functional binders. Previous studies have shown that such metrics effectively identify true negatives, but carry a high false-positive rate, limiting practical utility (68). Our tiered structure quality assessment of TCR-pMHC models can be adapted to other protein-protein interaction systems and could improve the success rate of designed molecules by enabling a more fine-grained and comprehensive quality stratification, thereby reducing the number of non-functional or irrelevant candidates prioritized for experimental testing.

Despite the progress in high-confidence TCR-pMHC structural modeling and quality assessment, several limitations remain. High-confidence predictions are constrained by the intrinsic flexibility of CDR loops, which are difficult to model accurately in AF3. Additionally, the quasi-rigid treatment of complexes, restricts the capture of dynamic conformational states critical for pMHC recognition (69). Future work could integrate molecular dynamics simulations or multiple AF3 models generated from different diffusion seeds to better sample conformational states, as recent studies suggest that AF3 can capture alternative binding conformations (70, 71). Along with this, our framework relies on confidence metrics derived from predicted structures rather than direct energetic calculations, meaning that structural plausibility does not necessarily equate to binding affinity to pMHCs or functional potency of the resulting TCR interaction. While energetic evaluations could provide complementary information, including them would substantially reduce throughput and increase computational cost, limiting the ability to perform large-scale assessments of thousands of TCR-pMHC models efficiently. Furthermore, the training dataset, although carefully curated, remains limited to the modest size and diversity of experimentally resolved TCR-pMHC complexes, which may bias the classifier toward canonical docking geometries and underrepresent unconventional binding modes. For this reason, models classified as low-quality (LQ) may not necessarily correspond to non-immunogenic interactions. They may still provide valuable structural insights, potentially representing binding modes that exist but are not yet captured in current TCR-pMHC experimental databases. Incorporating physics-based energy evaluations or ensemble-based scoring strategies could further refine the discrimination between non-functional immune interactions and actionable TCR-pMHC complexes.

Overall, our framework demonstrates that integrating structural modeling with data-driven quality assessment enhances the structural and functional interpretation of TCR-pMHC interactions. Beyond improving model selection, our random forest classifier provides a scalable approach to refine the generation of large TCR-pMHC structural datasets and filter out low-confidence models enriched in non-immunogenic TCR-pMHC interactions. Collectively, this framework can offer tangible benefits for both fundamental immunoinformatic research and translational applications in TCR-based immunotherapies.

## 4 Methods

### 4.1 Data retrieval

Experimentally resolved TCR-pMHC class I experimental structures were retrieved from the TCR3D (72) database, a curated collection of TCR-related structures derived from the Protein Data Bank (PDB (9)) (downloaded 1 January, 2026). We filtered for human and mouse complexes and excluded non-canonical docking modes, resulting in 265 TCR-pMHC complexes. In our analysis, potentially non-canonical docking geometries were identified after standardizing the orientation of the MHC molecule across structures. We used two structural criteria to flag extreme configurations that were not directly comparable with the canonical class I TCR-pMHC binding mode: (i) instances in which the TCR is oriented toward the underside of the MHC β-sheet or the peptide is positioned beneath the β-sheet, corresponding to configurations where the TCR and peptide are located on opposite sides of the MHC surface; and (ii) cases in which the TCR-MHC unit vector shows a negative x-component in the standardized coordinate frame, indicating orientations where the TCR CDR3 loops point away from the MHC or engage the complex via non-classical interfaces, such as interactions dominated by constant domains.

The TCRvdb was obtained from Messemaker et al., 2025 (50). It consist on VDJdb (49) entries of GLCTLVAML (GLC; Epstein-Barr virus, EBV) and YLQPRTFLL (YLQ; SARS-CoV-2) epitopes paired with multiple TCRs and restricted to HLA-A*02:01 that have been experimentally reevaluated using a harmonized T-cell validation system. Following the authors’ recommendations, a significance threshold of 1 × 10^−5^ was applied to define validating interactions.

The IMMREP23 (46) dataset was downloaded from the official GitHub repository: https://github.com/justin-barton/IMMREP23/ and contains TCR-pMHC annotated complexes spanning 20 epitopes with experimentally validated positives and synthetic negatives generated by swapping TCRs across unrelated pMHCs in a 1:5 positive to negative ratio.

In the TCRvdb and IMMREP23 datasets, full-length TCR sequences were reconstructed using Stitchr (73) based on annotated V and J gene segments and CDR3α/β sequences. This represents a standard procedure in TCR repertoire analysis in both computational and experimental studies (30, 46, 74, 75) and was applied here since sufficient V and J gene annotation information was available to enable deterministic reconstruction of full-length sequences. These entries were then submitted to ANARCI (76) for annotation of individual CDR regions using the IMGT numbering scheme (77). MHC sequences were retrieved from the IPD-IMGT/HLA database (77, 78) using the first available eight-digit entry (e.g., HLA-A01:01:01:01 for HLA-A01:01) as MHC alleles were annotated at four-digit resolution.

A complete summary of all datasets used in this study, including the number of complexes, unique TCRs, HLA restrictions, epitope diversity, and filtering criteria, is provided in **Supplementary Tables 1** and **2**.

### 4.2 Benchmarking of protein modelling tools on TCR-pMHC class I complexes

We benchmarked state-of-the-art protein structure modelling tools: four general-purpose methods, AlphaFold-Multimer v2.3 (28), AlphaFold3 (25), Chai-1 (26), Boltz-2 (27), and three TCR-specific tools, TCRmodel2 (29), tFold-TCR (30) and TCRdock (12).

These methods were selected to cover the main paradigms in protein complex prediction. AlphaFold-Multimer v2.3 (AF2.3) serves as a transformer-based baseline for multi-chain modeling, while AlphaFold3 (AF3) represents a diffusion-based generative successor extending this line. Chai-1 and Boltz-2 provide additional diffusion-based, open-weight generalist models for comparison. Three TCR-specific approaches were also included to assess the impact of domain specialization. TCRmodel2 is built on AlphaFold-Multimer but introduces TCR-pMHC-specific MSAs, targeted template selection, and curated pMHC structural templates to improve complex modeling. Similarly, TCRdock is based on an AlphaFold2.3 and performs TCR-pMHC docking using template-guided structural assembly, generating hybrid multichain templates derived from sequence-similar complexes. In contrast, tFold-TCR uses a protein language model fine-tuned on immune receptor sequences to capture TCR-pMHC-specific signals via an alternative, sequence-centric strategy. For TCRdock, only a subset of benchmark complexes could be generated due to limited template coverage of TCR genes and MHC alleles in its underlying databases (**Supplementary Table 3**).

We evaluated their performance on experimentally resolved TCR-pMHC class I structures from the PDB (9). To assess generalization capabilities consistently across methods, the dataset was stratified into pre- and post-cutoff subsets according to the model’s reported training cutoff date. To ensure an unbiased comparison, we applied both the latest training date as uniform cutoff across all methods (Boltz-2: 2023-06-01) and separately we studied the performance of each method using its specific training cutoff. The reported training cutoffs were: TCRmodel2 (2018–04–30), AlphaFold-Multimer v2.3, TCRdock and AlphaFold3 (2021–09–30), Chai-1 (2021–01–12), tFold-TCR (2022–01–01), and Boltz-2 (2023–06–01). The post-cutoff subsets for each modelling tool, based on their respective training cutoffs, are detailed in **Supplementary Table 3**. The post-cutoff IDs for Boltz-2 are: 8RLU, 9IKY, 9J4S, 9J4T, 9HLJ, 9GV7, 8V51, 8YIV, 8SHI, 8RYO, 8RYQ, 8RRO, 8RLV, 8RJ5, 8WUL, 8F5A, 8YE4, 8RYM, 9J4U, 9D95, 8I5D, 8EN8, 8V50, 9GV6, 8V4Z, 8QFY, 8I5C, 9RXM, 8EO8, 8YJ2, 8RLT, 8RYP, 8ENH, 9RU5, 8DNT, 8RYN, 9RUP, 8WTE, 9C3E.

All tools were run with default parameters using five input sequences as independent molecular chains: TCRα, TCRβ, peptide, MHC, and β2-microglobulin (β2M) unless stated otherwise. For TCRdock, a different input format was used, as its pipeline operates from a TSV file containing TRAV, TRAJ, TRBV, TRBJ genes, CDR3α/β sequences, and HLA alleles, from which sequences are retrieved from internal databases. TCRmodel2 and TCRdock do not support β2M input and were therefore run without including this chain. For PDB complexes, amino acid sequences were extracted directly from the corresponding annotated structures. For the IMMREP23 and TCRvdb datasets, TCR sequences were reconstructed using Stitchr (73), and MHC sequences retrieved from the IPD-IMGT/HLA (77, 78) database, as described in Section 4.1.

Protein chain assignments differed across tools: for AF3, AF2.3, Chai-1, and Boltz-2, chains were assigned as MHC (chain A), β2M (chain B), peptide (chain C), TCRα (chain D), and TCRβ (chain E); for tFold-TCR as TCRα (chain A), TCRβ (chain B), MHC (chain M), β2M (chain N), and peptide (chain P); for TCRmodel2 as MHC (chain A), peptide (chain C), TCRα (chain D), and TCRβ (chain E). For TCRdock, models were generated in a single-chain representation and subsequently decomposed into individual chains based on residue gaps between components, corresponding to MHC (chain A), peptide (chain C), TCRα (chain D), and TCRβ (chain E).

Importantly, both TCRdock and TCRmodel2 output only the TCR variable domains and the MHC peptide-binding groove, rather than full-length TCR or MHC chains. Five structural models were generated for each complex. No artificial linkers or bonds were introduced at the input stage for any of the tools evaluated. No chain ID normalization was applied across tools; instead, chain identity was tracked throughout the pipeline via explicit per-tool chain mappings, including the chain annotations from the original PDB structures.

Modelling was performed using a setup with 1 NVIDIA Hopper H100 GPU (64 GB HBM2) and 20 CPUs, with an average computation time per complex (5 models) of 1.5 minutes for Boltz-2, 2 minutes for tFold-TCR, 3.5 minutes for Chai-1, 10 minutes for TCRmodel2, 40 minutes for AF3, and 2.5 hours for AlphaFold-Multimer v2.3. MSAs generated by AF3 were provided as input to Boltz-2 and Chai-1.

### 4.3 Structural comparison metrics

Predicted models were evaluated against the corresponding experimental structures or compared with each other using multiple metrics: interface root means square deviation (iRMSD) calculated for the full interface and separately for the MHC chain, TCR chains, and peptide. The interface was defined as all atoms within 10 Å of the peptide chain. Heavy-atom RMSD was computed for CDR3α and CDR3β, and overall interface quality was assessed using the DockQ (55). Docking geometries were analyzed using the TCRdock software (12) which assigns six rigid-body orientation parameters to each model, and visualized using principal component analysis (PCA).

Models were classified based on two representative metrics: TCR chain iRMSD (TCR iRMSD) and DockQ score as follows: high quality (HQ; TCR-iRMSD ≤ 2 Å and DockQ ≥ 0.8), medium quality (MQ; TCR-iRMSD ≤ 5 Å and DockQ ≥ 0.49), acceptable quality (AQ; TCR-iRMSD < 5 Å and DockQ ≥ 0.23), and low quality (LQ; TCR-iRMSD > 5 Å or DockQ < 0.23). Consensus analysis across replicas was performed by comparing models pairwise using TCR-iRMSD and DockQ scores.

### 4.4 Model quality metrics

Intrinsic model confidence metrics were extracted to enable reference-free quality assessment, including several levels of structural assessment: structural confidence measured using predicted local distance difference test scores (pLDDT) for the full complex and for subsets focusing on CDRα/β regions; protein-protein interface confidence metrics such as the interface predicted TM-score (ipTM) (35) and a TCR-pMHC-specific ipTM (29), the average interface predicted aligned error (iPAE) scaled to yield a dimensionless confidence score (39), the average interface predicted distance error (iPDE) (36) and the interaction prediction score from aligned errors (ipSAE) (37). In addition, we evaluated the predicted DockQ scores v1 (38) and v2 (39), computed specifically for the TCR-pMHC interface.

### 4.5 Structural annotation and model processing

Chains of the PDB structures were annotated using the script “mir-1.0-SNAPSHOT.jar” provided by Karnaukov et al. (14) which outputs a general.txt file containing the correspondence between chain type and chain ID for each PDB complex. Model chains were annotated during modeling as described in section 4.2.

To calculate TCR-pMHC-specific interface metrics (DockQ, iPAE, iPDE, pDockQ, and pDockQ2 scores), a *post-hoc* analysis was performed in which the independently modeled chains were merged such that chain A included MHC-I, β2-microglobulin, and the peptide, while chain B included TCRα and TCRβ. The pDockQ (38) and pDockQ2 (25) scripts were adapted to AlphaFold3 outputs. ipSAE was calculated using default parameters (37). pLDDT and ipTM scores were parsed directly from the AlphaFold3 modelling outputs. A TCR-pMHC-specific ipTM was computed as the average of the pairwise ipTM scores spanning the TCR-pMHC interface, specifically those involving TCRα-peptide, TCRβ-peptide, TCRα-MHC, and TCRβ-MHC chain pairs. PyMOL (79) was used for structural visualization and figure generation.

### 4.6 Sequence similarity analysis

Sequence similarity between post- and pre-cutoff TCR–pMHC complexes was computed at the level of CDR3 loops, epitopes, and MHC sequences. For each post-cutoff structure, similarity was evaluated against all pre-cutoff structures, and the maximum similarity value was retained per feature.

CDR3 loop and epitope similarity were computed using a normalized Levenshtein distance implemented in the python-Levenshtein package, defined as 1 - (edit distance/max sequence length), ensuring comparability across sequences of different lengths. This metric inherently accounts for insertions, deletions, and substitutions, and produces values in the range [0, 1], with 1 indicating identical sequences. CDR3α and β similarities were aggregated by averaging.

MHC sequences were compared using local sequence alignment with the Smith-Waterman algorithm implemented in Biopython (pairwise2 module). This algorithm performs local alignment without requiring global end-to-end matching, allowing partial alignment of variable-length sequences. The alignment used a scoring scheme of +2 for matches, -1 for mismatches, and gap opening and extension penalties of -5 and -0.5, respectively. The resulting alignment score was normalized by the theoretical maximum score (2 × min (sequence lengths)) to obtain a similarity measure bounded between 0 and 1.

### 4.7 Structural similarity analysis

Structural similarity between post-cutoff and pre-cutoff TCR-pMHC complexes was assessed using DockQ scores and TCR interface RMSD (TCR-iRMSD) (computed as described in Section 4.3). For each post-cutoff structure, all-against-all comparisons to pre-cutoff structures were performed, and the maximum DockQ and minimum TCR-iRMSD values were retained as measures of closest structural similarity.

These similarity analyses were performed independently for two temporal cutoffs: (i) the unified Boltz-2 cutoff (2023-06-01; n = 39 post-cutoff structures), and (ii) the AlphaFold3-specific cutoff (2021-09-30; n = 88 post-cutoff structures), enabling assessment of robustness across different training-time definitions.

### 4.8 Exhaustive metric combinations

Individual confidence metrics were combined using Boolean operators (AND/OR) and literature-established thresholds to evaluate a total of 265,720 combinations across three quality transitions: LQ vs. AQ/MQ/HQ, LQ/AQ vs. MQ/HQ, and LQ/AQ/MQ vs. HQ. Acceptability thresholds were set at 70 for pLDDTs, 0.23 for pDockQ and pDockQ2, and 0.6 for ipTM and ipSAE. Medium-quality thresholds were defined as 0.49 for pDockQ and pDockQ2. High-quality thresholds were set at 90 for pLDDT, and 0.8 for ipTM, ipSAE, pDockQ, and pDockQ2. These thresholds were applied to the following default model confidence metrics: full-complex pLDDT, CDRα and CDRβ pLDDT, mean ipTM, TCR-pMHC-specific ipTM, ipSAE, and predicted DockQ scores (v1 and v2).

Filters were derived from 265 PDB-modeled complexes (1,325 models) using a five-fold cross-validation scheme, with performance evaluated on the corresponding test sets and reported as the mean across the five folds. Train-test splits were designed so that no models from the same complex were shared between folds. Filters were optimized either for maximum precision and specificity or for a balance between specificity and recall, prioritizing higher-quality models while minimizing false positives and maintaining relatively high recall. Classification of models into quality tiers was determined based on whether each model passed the corresponding defined filters.

### 4.9 Random forest classifier

The random forest classifier was trained on 265 PDB (9) complexes modelled using AlphaFold3 (25) generating five model replicas per TCR-pMHC complex (1,325 models in total). Ground-truth quality labels were derived from model versus experimental comparison metrics applied to experimentally resolved PDB structures (See Methods, Section 4.3). Input features consisted of reference-independent model confidence metrics, ensuring a reference-free training space (See Methods, Section 4.4).

Training was performed using scikit-learn with default parameters and evaluated through a stratified 80/20 train-test split repeated over 100 random seeds (80). Data splits were designed so that no models from the same complex appeared in both training and test sets, preventing data leakage. Feature importance was quantified using Shapley additive values (SHAP) across the 100 train/test splits (56).

### 4.10 Statistical analysis

Association analyses between quality tiers and validating/positive TCR-pMHC interactions were performed using the Chi-square (χ²) test, and Odds Ratios (OR) were computed separately for the positive and negative classes to quantify the strength of association.

Receiver Operating Characteristic (ROC) curves and the corresponding Area Under the Curve (AUC ROC) values were evaluated using the DeLong test to compare predictive performance across models. Precision-Recall (PR) curves and the associated Area Under the Curve (AUC PR) values were assessed using bootstrap resampling.

Bonferroni correction was applied to account for multiple comparisons in both cases. Correlations were measured using Spearman’s rank correlation coefficient. Significance thresholds were adjusted accordingly, with an adjusted p-value < 0.05 considered statistically significant.

## Data Availability

The IMMREP23 and PDB TCR-pMHC class I AF3-modeled datasets generated and analyzed in this study are available at https://huggingface.co/datasets/Alexasparis/AF3TCRpMHC. The code and repository can be accessed at https://github.com/Alexasparis/AF3TCRpMHC.git. The TCRvdb dataset is originally from Messemaker et al. (50) and the official GitHub repository is available at https://github.com/schumacherlab/TCRvdb.git.
