# Supplementary material for: Structural quality-tier assessment for TCR-pMHC functional enrichment
Source: Front Immunol. 2026 Jul 24;17:1869810. doi: 10.3389/fimmu.2026.1869810 (PMC13447355; doi:10.3389/fimmu.2026.1869810)
Supplement: Supplementary file 1 [file DataSheet1.pdf]

Supplementary Material

1. Supplementary Tables

Supplementary Table 1. Summary of datasets used throughout the study (I). The table reports the number of total TCR-pMHC complexes (N. Complexes), generated structural models (N. Models), unique TCRs, distinct MHC alleles, and unique epitopes included in each dataset. The "Purpose" column indicates the intended use of each dataset, including benchmarking of protein modelling tools and Random Forest (RF) model development, as well as RF applications using either balanced (~1:1 positive to negatives) or imbalanced (~1:5 positives to negatives) class compositions. For the benchmark dataset, the applied filtering criteria are also indicated. PDB pre and PDB post refer to the split according to the training cutoff of the most stringent modelling tool (Boltz-2) used for the benchmark.

Dataset	N. Complexes	N. Models	Unique TCRs	MHC-I alleles	Epitopes	Purpose	Filtering
PDB pre	226	1,130	128	33	136	Benchmark and RF development	Filter non-canonical docking poses and non-human/mouse complexes
PDB post	39	195	29	15	25	Benchmark and RF development	
TCRvdb	606	3,030	606	1	2	RF application (positives and negatives ~1:1)	Filter non-canonical docking poses
IMMREP23	3484	17,420	729	6	20	RF application (positives and negatives ~1:5)	

Supplementary Table 2. Summary of datasets used throughout the study (II). The table reports the diversity of T-cell receptor (TCR) components and antigens across all datasets used for benchmarking and model training/evaluation. Specifically, it includes the number of unique TRAV, TRAJ, TRBV, and TRBJ gene segments, as well as the distributions of CDR3α and CDR3β lengths. MHC class I allele diversity and epitope length distributions are also reported where applicable. Values are shown as frequency distributions (length:count or category counts). This overview highlights differences in repertoire composition across datasets used for structure prediction benchmarking (PDB pre and PDB post) and machine learning applications (TCRvdb and IMMREP23), reflecting variability in receptor diversity, antigen representation, and class imbalance across data sources.

Dataset	TRAV genes	TRAJ genes	TRBV genes	TRBJ genes	CDR3a lengths	CDR3b lenghts	MHC alleles	Epitope lengths
PDB pre	47	39	40	12	15:76, 14:44, 13:44, 12:30, 11:12	15:76, 14:44, 13:44, 12:30, 11:12	H2-Kb, H2-Db, H2-Ld, B*27:05, A*02:01, A*03:01, A*24:02, B*35:01, E*01:130, A*02:823, B*35:08, B*07:02, B*37:01, B*08:01, A*02:614, A*11:01, B*51:01, C*08:02, A*02:1, H2-Dd, B*57:01, A*01:01, E*01:150, B*53:01, B*44:05, B*27:255, H2-Kbm8, H2-Kbm3, B*42:01, C*05:01, B*81:14, A*02:06	9:128, 10:46, 8:14, 11:9, 13:7, 12:1, 4:1
PDB post	18	20	28	20	15:21, 13:12, 14:12, 12:11, 11:6, 8:1	15:32, 14:11, 11:10, 13:8, 12:2	A*11:01, A*03:01, B*38:01, B*35:01, E*01:150, A*01:01, A*02:01, B*57:01, A*01, A*24:02, B*07, E*01:03, B*07:01, C*12:01, C*06:02	9:45, 10:13, 8:4, 11:1
TCRvdb	44	53	49	13	9:3, 10:30, 11:216, 12:68, 13:95, 14:91, 15:46, 16:35, 17:15, 18:4, 19:2, 20:1	10:1, 11:53, 12:20, 13:228, 14:106, 15:82, 16:88, 17:17, 18:6, 19:5	A*02:01	9:606
IMMREP23	57	55	52	14	5:2, 6:6, 7:10, 8:54, 9:305, 10:570, 11:647, 12:796, 13:622, 14:275, 15:145, 16:36, 17:8, 18:5, 19:1, 22:2	7:25, 8:9, 9:58, 10:213, 11:813, 12:739, 13:675, 14:544, 15:260, 16:75, 17:45, 18:7, 19:19, 20:2	A*01:01, A*02:01, A*11:01, B*07:02, B*08:01, B*35:01	8:362, 9:2396, 10:324, 11:402

Supplementary Table 3. Post-training cutoff PDB IDs per TCR-pMHC modelling tool. Summary of the post training cutoff sets for each structure prediction method. For each tool, the specific training cutoff date is indicated alongside the total number (N. Post PDB IDs) and the explicit list of protein structures (PDB IDs) deposited after that date.

Modeling Tool	Cutoff Date	N. Post PDB IDs	Post PDB IDs
Boltz-2	2023-06-01	39	8RLU, 9IKY, 9J4S, 9J4T, 9HLJ, 9GV7, 8V51, 8YIV, 8SHI, 8RYO, 8RYQ, 8RRO, 8RLV, 8RJ5, 8WUL, 8F5A, 8YE4, 8RYM, 9J4U, 9D95, 8I5D, 8EN8, 8V50, 9GV6, 8V4Z, 8QFY, 8I5C, 9RXM, 8EO8, 8YJ2, 8RLT, 8RYP, 8ENH, 9RU5, 8DNT, 8RYN, 9RUP, 8WTE, 9C3E
tFold-TCR	2022-01-01	85	8RLU, 8GVG, 7PBC, 8GOM, 7NA5, 8RYO, 7PB2, 7RM4, 7Q99, 6ZKX, 8YE4, 8V50, 9D95, 7Q9B, 7PDW, 7NDT, 7N2Q, 7Q9A, 7NMF, 8YIV, 8RLV, 8RJ5, 7NME, 8RYM, 9J4U, 7QPJ, 8QFY, 7L1D, 6ZKY, 7DZM, 9RXM, 7PBE, 8GVI, 8GVB, 7N5P, 7PHR, 7SU9, 7NMG, 9J4S, 7OW6, 7N5C, 9HLJ, 8GON, 8SHI, 8V51, 8RRO, 8RYQ, 8F5A, 8I5D, 8EN8, 7DZN, 7NDQ, 7RK7, 6ZKW, 7RRG, 8YJ2, 8RYP, 8CX4, 8RYN, 8WTE, 9C3E, 7NDU, 8D5Q, 7N4K, 7N2N, 9IKY, 7N2S, 9J4T, 9GV7, 7N2O, 8WUL, 8V4Z, 9GV6, 8I5C, 7N2P, 8EO8, 7OW5, 8RLT, 7N2R, 7R80, 8ENH, 9RU5, 8DNT, 9RUP, 6ZKZ
Chai-1	2021-01-12	98	8RLU, 8GVG, 7PBC, 8GOM, 7RTR, 7NA5, 8RYO, 7PB2, 7RM4, 7Q99, 6ZKX, 8YE4, 9D95, 8V50, 7Q9B, 7PDW, 7NDT, 7N2Q, 7Q9A, 7NMF, 8ES8, 7JWJ, 8YIV, 8RLV, 8RJ5, 7NME, 8RYM, 9J4U, 7QPJ, 6VMC, 8QFY, 7L1D, 6ZKY, 7DZM, 9RXM, 7PBE, 6VMA, 6VM9, 8GVI, 7N1F, 8GVB, 7N5P, 7PHR, 7BYD, 7SU9, 7NMG, 9J4S, 7OW6, 7N5C, 9HLJ, 8GON, 8V51, 8SHI, 8RYQ, 8RRO, 8F5A, 8I5D, 8EN8, 7DZN, 7NDQ, 7RK7, 6ZKW, 7RRG, 8YJ2, 8RYP, 8ES9, 8CX4, 8RYN, 8WTE, 9C3E, 7NDU, 7N4K, 8D5Q, 6VM7, 7N2N, 9IKY, 7N2S, 9J4T, 9GV7, 7N2O, 8WUL, 8V4Z, 9GV6, 8I5C, 7N2P, 8EO8, 7OW5, 8RLT, 7R80, 7N2R, 8ENH, 7JWI, 9RU5, 8DNT, 9RUP, 7N1E, 7N6E, 6ZKZ
AF3/AF2.3	2021-09-30	88	8RLU, 8GVG, 7PBC, 8GOM, 7RTR, 7NA5, 8RYO, 7PB2, 7RM4, 7Q99, 6ZKX, 8YE4, 9D95, 8V50, 7Q9B, 7PDW, 7NDT, 7N2Q, 7Q9A, 7NMF, 8ES8, 8YIV, 8RLV, 8RJ5, 7NME, 8RYM, 9J4U, 7QPJ, 8QFY, 7L1D, 6ZKY, 7DZM, 9RXM, 7PBE, 8GVI, 8GVB, 7N5P, 7PHR, 7SU9, 7NMG, 9J4S, 7OW6, 7N5C, 9HLJ, 8GON, 8V51, 8SHI, 8RRO, 8RYQ, 8F5A, 8I5D, 8EN8, 7DZN, 7NDQ, 7RK7, 6ZKZ, 7RRG, 8YJ2, 8RYP, 8ES9, 8CX4, 8RYN, 8WTE, 9C3E, 7NDU, 7N4K, 8D5Q, 7N2N, 9IKY, 7N2S, 9J4T, 9GV7, 7N2O, 8WUL, 8V4Z, 9GV6, 8I5C, 7N2P, 8EO8, 7OW5, 8RLT, 7R80, 7N2R, 8ENH, 9RU5, 8DNT, 9RUP
TCRdock	2021-09-30	63	8RLU, 7NMG, 7N2N, 7N2S, 8GVG, 7PBC, 7OW6, 7N5C, 9GV7, 7RTR, 8V51, 7N2O, 8SHI, 8RYO, 7PB2, 7RM4, 8RYQ, 7Q99, 8RJ5, 8RLV, 7NME, 8F5A, 8YE4, 8RYM, 9J4U, 9D95, 8V50, 8EN8, 7Q9B, 7QPJ, 7DZN, 9GV6, 7L1D, 7PDW, 7N2P, 9RXM, 7RK7, 7RRG, 7PBE, 8EO8, 7OW5, 8V4Z, 8RLT, 8RYP, 8GVI, 8ES9, 7Q9A, 7R80, 7N2R, 8ENH, 8GVB, 9RU5, 8CX4, 8DNT, 8RYN, 9RUP, 7N5P, 9C3E, 7PHR, 7N4K, 8D5Q, 7NMF, 7SU9
TCRmodel2	2018-04-30	131	8RLU, 6VRN, 6VMX, 8GVG, 7PBC, 6EQA, 8GOM, 7RTR, 7NA5, 8RYO, 7PB2, 7RM4, 7Q99, 6ZKX, 5YXU, 8YE4, 9D95, 8V50, 7Q9B, 7PDW, 6RPA, 7NDT, 7N2Q, 7Q9A, 6AMU, 7NMF, 6MTM, 8ES8, 6L9L, 6RPB, 7JWJ, 8YIV, 6UON, 8RLV, 8RJ5, 7NME, 6RSY, 8RYM, 9J4U, 7QPJ, 6VMC, 8QFY, 7L1D, 6ZKY, 7DZM, 6UZ1, 9RXM, 6G9Q, 7PBE, 6VMA, 6VM9, 8GVI, 7N1F, 6ULR, 8GVB, 7N5P, 7PHR, 7SU9, 7NMG, 6Q3S, 9J4S, 7OW6, 7N5C, 9HLJ, 8GON, 8V51, 8SHI, 8RYQ, 8RRO, 6ULN, 8F5A, 8I5D, 8EN8, 7DZN, 6TMO, 6P64, 6D78, 7NDQ, 7RK7, 6ZKW, 7RRG, 6R2L, 8YJ2, 8RYP, 8ES9, 6EQB, 6AM5, 8CX4, 8RYN, 8WTE, 9C3E, 7NDU, 7N4K, 8D5Q, 6VM7, 7N2N, 9IKY, 7N2S, 6TRO, 9J4T, 5NHT, 6BJ2, 9GV7, 5NQK, 7N2O, 8WUL, 8V4Z, 6VRM, 9GV6, 6BJ3, 8I5C, 7N2P, 6BJ8, 8EO8, 7OW5, 6VQO, 8RLT, 6DKP, 7R80, 7N2R, 8ENH, 7JWI, 9RU5, 8DNT, 9RUP, 7N1E, 7N6E, 6ZKZ, 5YXN, 6VM8, 6RP9

2. Supplementary Figures:

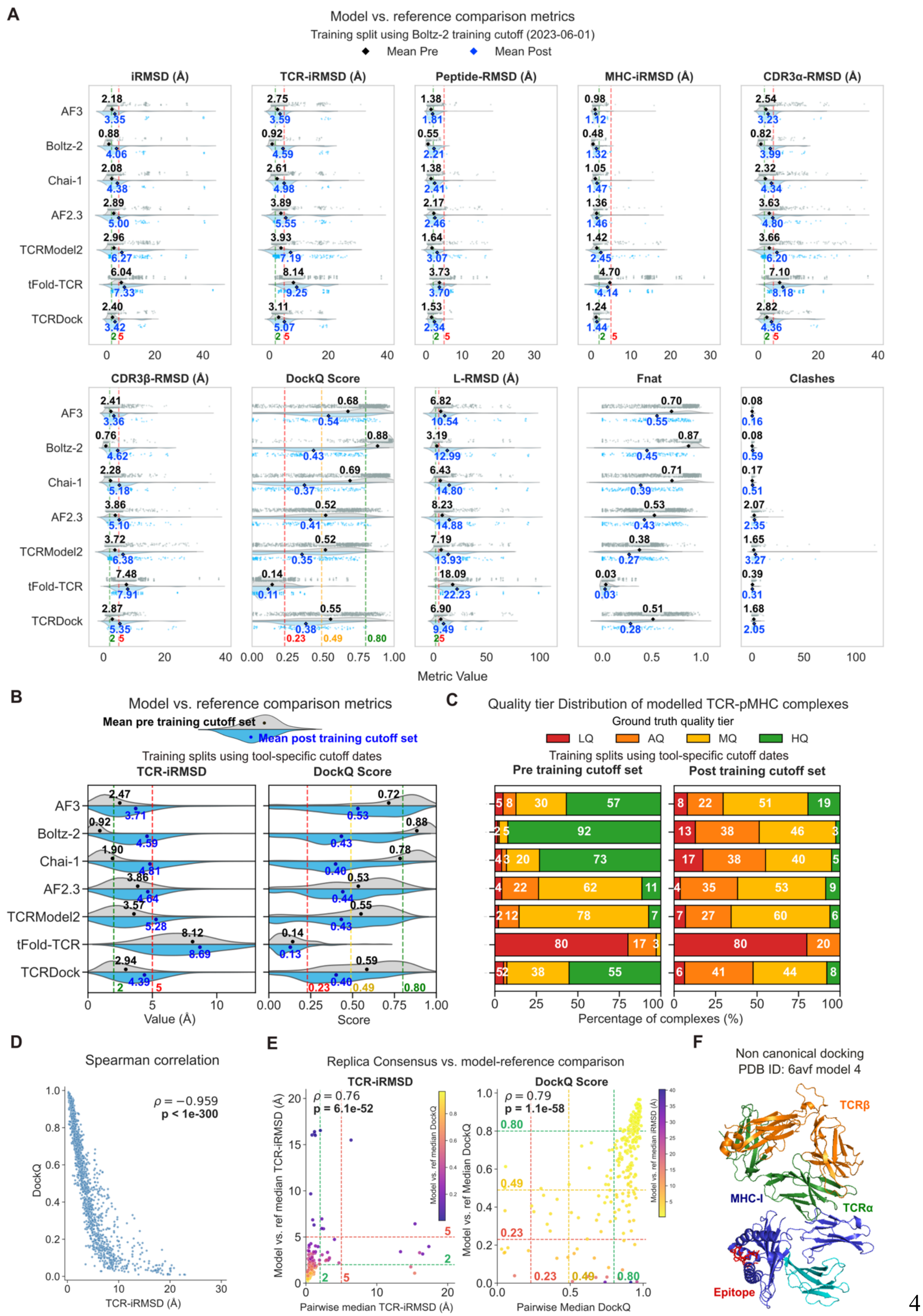

Supplementary Figure 1. Benchmarking of TCR-pMHC structure prediction tools (A) Distributions of model vs. experimental reference comparison metrics of TCR-pMHC class I complexes. Models were generated using state-of-the-art structure prediction tools, including general-purpose tools (AlphaFold3, Chai-1, Boltz-2, and AlphaFold-Multimer 2.3) and TCR-specific tools (TCRmodel2, tFold-TCR and TCRdock). Structures were divided based on deposition date in the PDB and the training date cutoff: before the training cutoff date (pre, gray) and after the training cutoff date (post, blue). For comparability, we applied the most stringent cutoff (Boltz-2: 2023-06-01) across all methods. Dashed lines indicate established thresholds for structural quality classification. Metrics include overall RMSD, TCR α/β interface RMSD (TCR-iRMSD), peptide RMSD, MHC interface RMSD, CDR3α RMSD, CDR3β RMSD, DockQ score, ligand RMSD (L-RMSD), fraction of native contacts (Fnat), and clashes. Numbers indicate the mean values of the metrics for each test set (pre: black, post: blue). (**B**) Distributions of TCR α/β interface RMSD (TCR-iRMSD) and DockQ scores for model vs. experimental reference structure comparisons of TCR-pMHC class I complexes. Models were generated using state-of-the-art structure prediction tools, including general-purpose (AlphaFold3, Chai-1, Boltz-2, and AlphaFold-Multimer 2.3) and TCR-specific tools (TCRmodel2, tFold-TCR and TCRdock). Structures were divided based on deposition date in the PDB and the training date cutoff from each modelling tool: before the training cutoff date (pre, gray) and after the training cutoff date (post, blue). The reported training cutoffs were: TCRmodel2 (2018-04-30), AlphaFold-Multimer v2.3, TCRdock and AlphaFold3 (2021-09-30), Chai-1 (2021-01-12), tFold-TCR (2022-01-01), and Boltz-2 (2023-06-01). Numbers indicate the mean values of the metrics for each set. Dashed lines indicate established thresholds for structural quality classification. (**C**) Percentages of HQ (high quality), MQ (medium quality), AQ (acceptable quality), and LQ (low quality) assigned to the highest quality model per complex among the five generated AF3 replicas per TCR-pMHC complex. Left panel shows the pre-training cutoff set, while right panel shows the post-training cutoff set. **(D)** Correlation between TCR-iRMSD and DockQ scores. ρ indicates the Spearman correlation coefficient, and the corresponding p-value is shown. **(E)** Correlations between median pairwise model replica comparisons and median model vs. reference DockQ scores, as well as TCR-iRMSD values. ρ indicates the Spearman correlation coefficient, and the corresponding p-value is shown. Colors represent the values of the complementary metric. **(F)** Example of a TCR-pMHC model classified having a non-canonical binding mode in the docking geometry analysis, where the TCR is not properly oriented towards the pMHC.

A

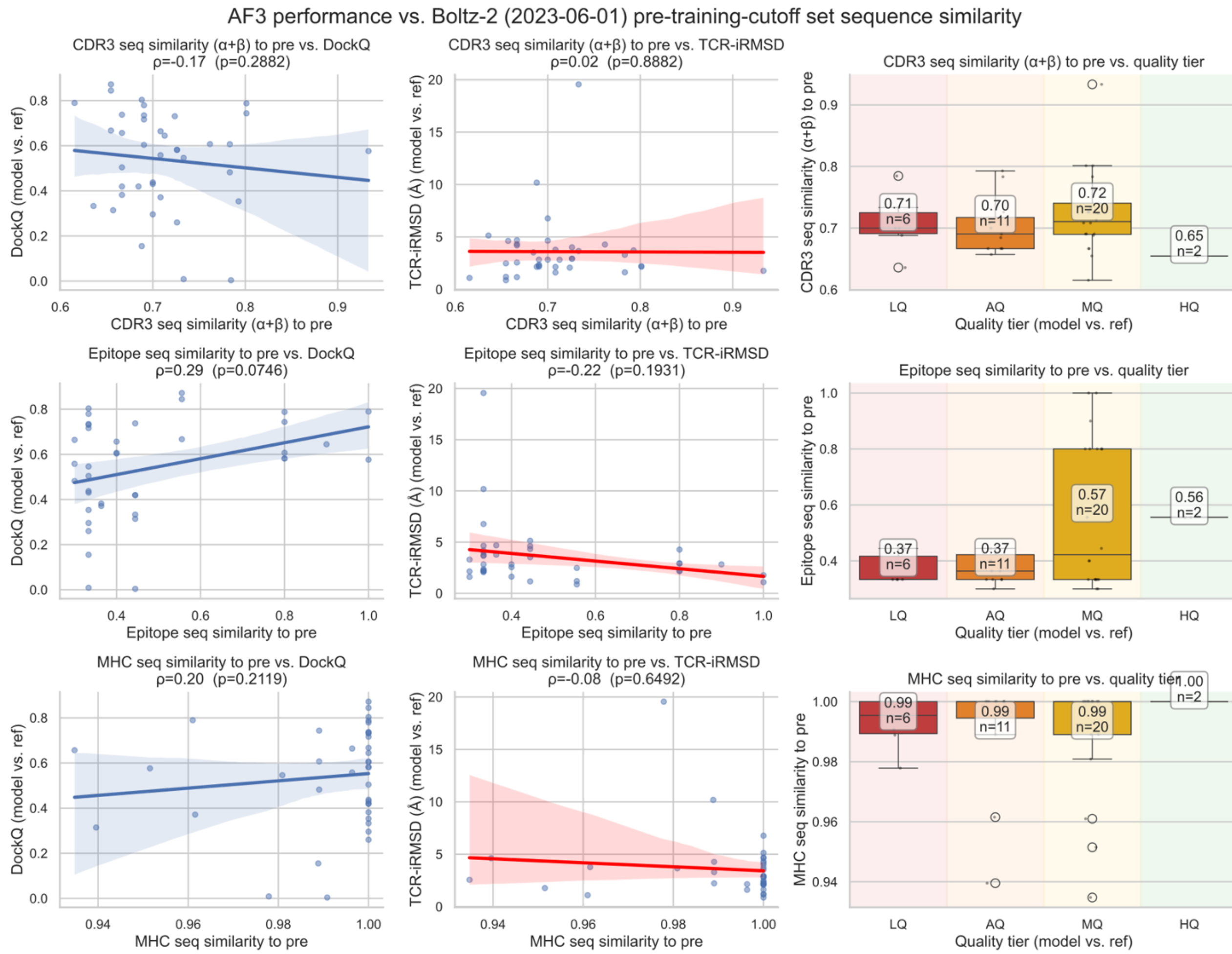
AF3 performance vs. Boltz-2 (2023-06-01) pre-training-cutoff set sequence similarity
CDR3 seq similarity (α+β) to pre vs. DockQ
ρ=-0.17 (p=0.2882)
CDR3 seq similarity (α+β) to pre vs. TCR-iRMSD
ρ=0.02 (p=0.8882)
CDR3 seq similarity (α+β) to pre vs. quality tier
Epitope seq similarity to pre vs. DockQ
ρ=0.29 (p=0.0746)
Epitope seq similarity to pre vs. TCR-iRMSD
ρ=-0.22 (p=0.1931)
Epitope seq similarity to pre vs. quality tier
MHC seq similarity to pre vs. DockQ
ρ=0.20 (p=0.2119)
MHC seq similarity to pre vs. TCR-iRMSD
ρ=-0.08 (p=0.6492)
MHC seq similarity to pre vs. quality tier
DockQ (model vs. ref)
TCR-iRMSD (Å) (model vs. ref)
CDR3 seq similarity (α+β) to pre
Epitope seq similarity to pre
MHC seq similarity to pre
Quality tier (model vs. ref)
LQ
AQ
MQ
HQ

B

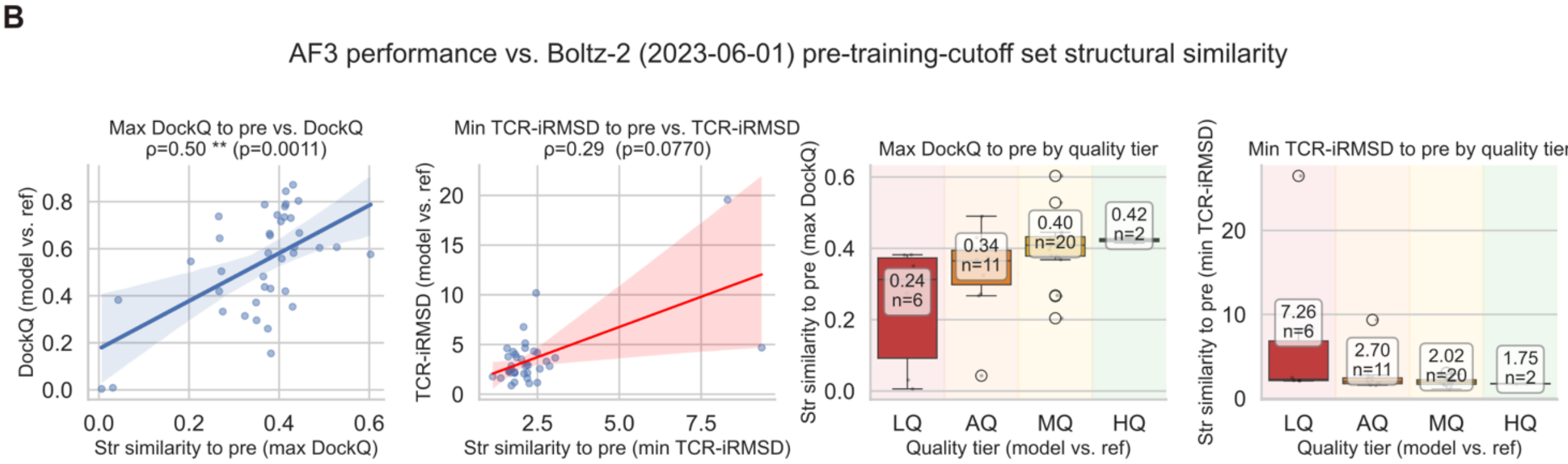
AF3 performance vs. Boltz-2 (2023-06-01) pre-training-cutoff set structural similarity
Max DockQ to pre vs. DockQ
ρ=0.50 ** (p=0.0011)
Min TCR-iRMSD to pre vs. TCR-iRMSD
ρ=0.29 (p=0.0770)
Max DockQ to pre by quality tier
Min TCR-iRMSD to pre by quality tier
DockQ (model vs. ref)
TCR-iRMSD (model vs. ref)
Str similarity to pre (max DockQ)
Str similarity to pre (min TCR-iRMSD)
Quality tier (model vs. ref)
LQ
AQ
MQ
HQ

Supplementary Figure 2. Pre- versus post-training Boltz-2 cut-off: Relationship between structural and sequence similarity and TCR-pMHC complex modelling accuracy for AlphaFold3 models. Pair-wise sequence and structural similarity between post-training cutoff structures and the pre-training-cutoff set were computed using the cutoff date defined by the most recent tool included in the benchmark, Boltz-2 (2023-06-01). We then selected the most similar pairs and compared against AF3 modelling quality metrics obtained on post cut-off structures (DockQ and TCR-iRMSD of models vs. reference comparisons). (**A**) Sequence similarity analysis. For each post structure, maximum sequence similarity to the pre cutoff set was calculated separately for the CDR3α+β chains, the epitope, and the MHC. Each row shows Spearman correlations against DockQ (col. 1) and TCR-iRMSD (col. 2), and distribution across quality tiers, Low (LQ), Acceptable (AQ), Medium (MQ), and High (HQ) (col. 3). (**B**) Structural similarity analysis. Maximum DockQ and minimum TCR-iRMSD to the closest pre-cutoff structure are shown against model vs. reference quality metrics (TCR-iRMSD and DockQ) and stratified by quality tier (col. 3-4). Spearman's ρ and associated p-values are reported in each scatter plot title; significance thresholds: $^{*}p < 0.05$, $^{**}p < 0.01$, $^{***}p < 0.001$.

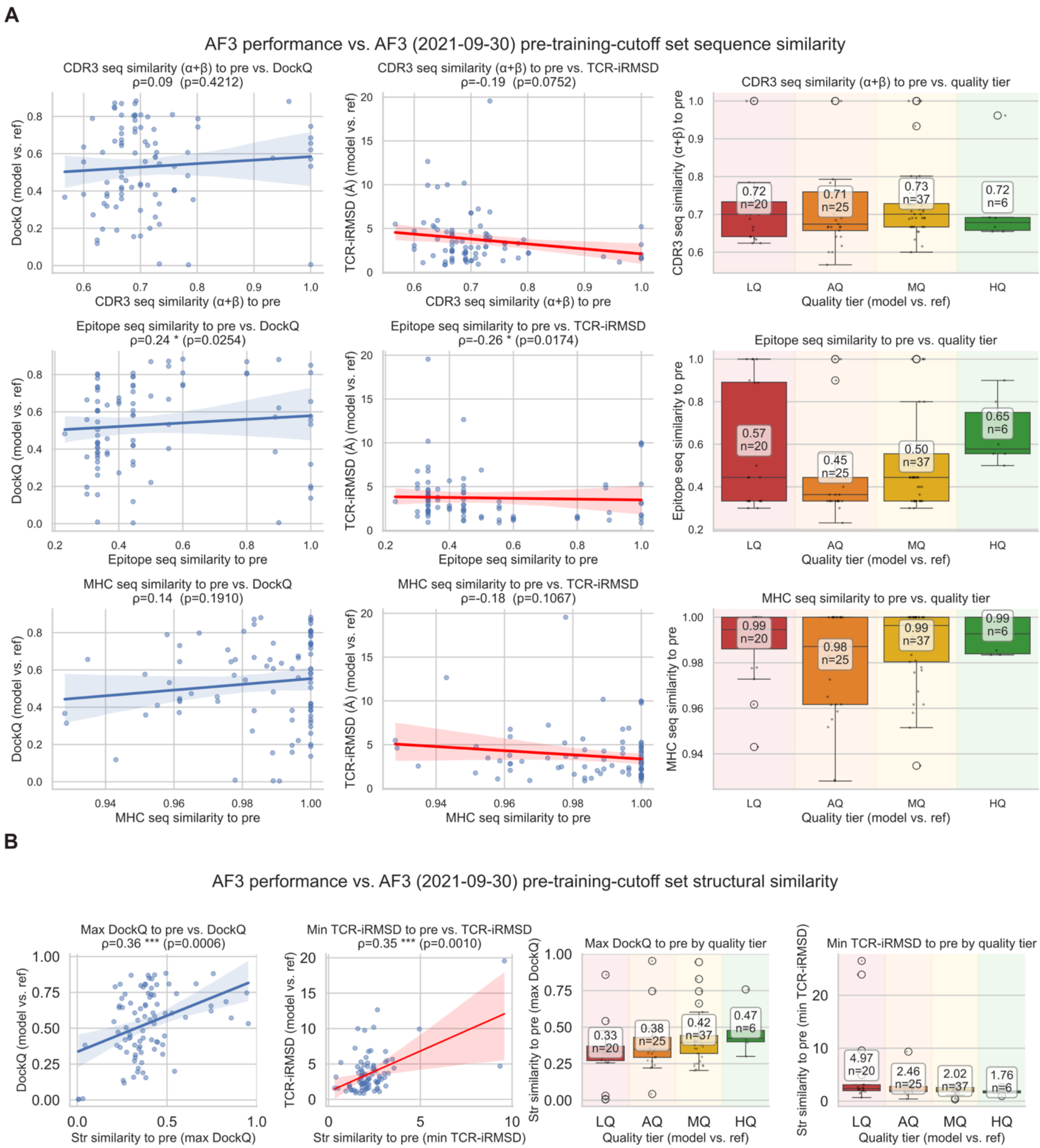

A
AF3 performance vs. AF3 (2021-09-30) pre-training-cutoff set sequence similarity
CDR3 seq similarity (α+β) to pre vs. DockQ
ρ=0.09 (p=0.4212)
DockQ (model vs. ref)
CDR3 seq similarity (α+β) to pre
CDR3 seq similarity (α+β) to pre vs. TCR-iRMSD
ρ=-0.19 (p=0.0752)
TCR-iRMSD (Å) (model vs. ref)
CDR3 seq similarity (α+β) to pre vs. quality tier
CDR3 seq similarity (α+β) to pre
0.72 n=20
0.71 n=25
0.73 n=37
0.72 n=6
LQ AQ MQ HQ
Quality tier (model vs. ref)
Epitope seq similarity to pre vs. DockQ
ρ=0.24 * (p=0.0254)
Epitope seq similarity to pre
Epitope seq similarity to pre vs. TCR-iRMSD
ρ=-0.26 * (p=0.0174)
Epitope seq similarity to pre vs. quality tier
0.57 n=20
0.45 n=25
0.50 n=37
0.65 n=6
MHC seq similarity to pre vs. DockQ
ρ=0.14 (p=0.1910)
MHC seq similarity to pre
MHC seq similarity to pre vs. TCR-iRMSD
ρ=-0.18 (p=0.1067)
MHC seq similarity to pre vs. quality tier
0.99 n=20
0.98 n=25
0.99 n=37
0.99 n=6
B
AF3 performance vs. AF3 (2021-09-30) pre-training-cutoff set structural similarity
Max DockQ to pre vs. DockQ
ρ=0.36 *** (p=0.0006)
DockQ (model vs. ref)
Str similarity to pre (max DockQ)
Min TCR-iRMSD to pre vs. TCR-iRMSD
ρ=0.35 *** (p=0.0010)
TCR-iRMSD (model vs. ref)
Str similarity to pre (min TCR-iRMSD)
Max DockQ to pre by quality tier
Str similarity to pre (max DockQ)
0.33 n=20
0.38 n=25
0.42 n=37
0.47 n=6
Min TCR-iRMSD to pre by quality tier
Str similarity to pre (min TCR-iRMSD)
4.97 n=20
2.46 n=25
2.02 n=37
1.76 n=6
LQ AQ MQ HQ
Quality tier (model vs. ref)

Supplementary Figure 3. Pre- versus post-training AF3 cut-off: Relationship between structural or sequence similarity and TCR-pMHC complex modelling accuracy in AlphaFold3 models. Pair-wise sequence and structural similarity between post-training cutoff structures and the pre-training-cutoff set were computed using the AF3 cutoff date (2021-09-30). We then selected the most similar pairs and compared against AF3 modelling quality metrics obtained on post cut-off structures (DockQ and TCR-iRMSD of models vs. reference comparisons). (**A**) Sequence similarity analysis. For each post structure, maximum sequence similarity to the pre cutoff set was calculated separately for the CDR3α+β chains, the epitope, and the MHC. Each row shows Spearman correlations against DockQ (col. 1) and TCR-iRMSD (col. 2), and distribution across quality tiers, Low (LQ), Acceptable (AQ), Medium (MQ), and High (HQ) (col. 3). (**B**) Structural similarity analysis. Maximum DockQ and minimum TCR-iRMSD to the closest pre-cutoff structure are shown against model vs. reference quality metrics (TCR-iRMSD and DockQ) and stratified by quality tier (col. 3-4). Spearman's ρ and associated p-values are reported in each scatter plot title; significance thresholds: $*p < 0.05$, $**p < 0.01$, $***p < 0.001$.

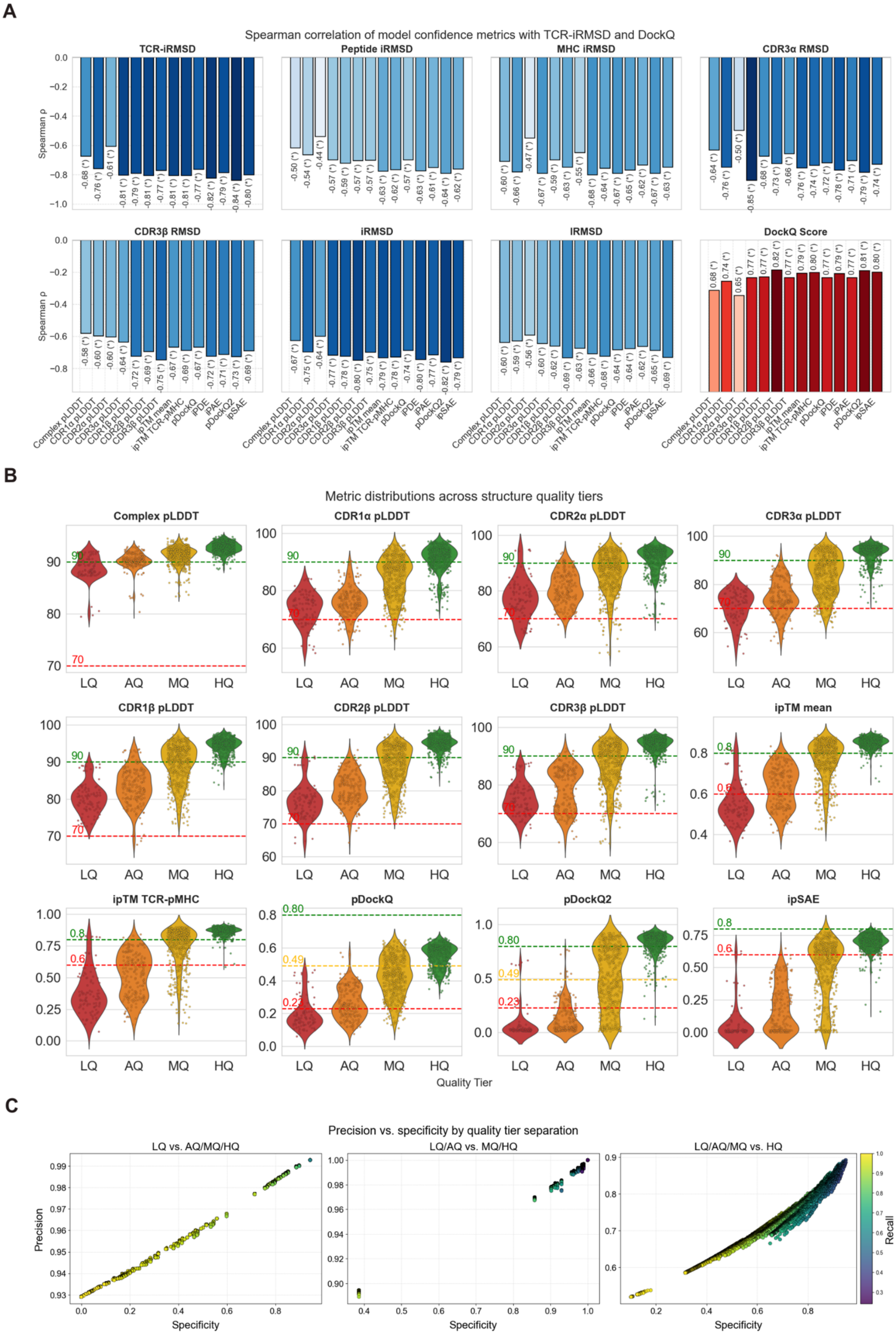
A
Spearman correlation of model confidence metrics with TCR-iRMSD and DockQ
TCR-iRMSD
Peptide iRMSD
MHC iRMSD
CDR3α RMSD
CDR3β RMSD
iRMSD
lRMSD
DockQ Score
Spearman ρ
Complex pLDDT
CDR1α pLDDT
CDR2α pLDDT
CDR3α pLDDT
CDR1β pLDDT
CDR2β pLDDT
CDR3β pLDDT
ipTM mean
ipTM TCR-pMHC
pDockQ
iPDE
iPAE
pDockQ2
ipSAE
B
Metric distributions across structure quality tiers
Complex pLDDT
CDR1α pLDDT
CDR2α pLDDT
CDR3α pLDDT
CDR1β pLDDT
CDR2β pLDDT
CDR3β pLDDT
ipTM mean
ipTM TCR-pMHC
pDockQ
pDockQ2
ipSAE
LQ
AQ
MQ
HQ
Quality Tier
C
Precision vs. specificity by quality tier separation
LQ vs. AQ/MQ/HQ
LQ/AQ vs. MQ/HQ
LQ/AQ/MQ vs. HQ
Precision
Specificity
Recall

Supplementary Figure 4. Evaluation of AF3 TCR-pMHC Class I structural model quality using model confidence metrics. **(A)** Spearman correlation coefficients between model confidence metrics and model vs. experimental reference comparison metrics. Model confidence metrics include full complex, CDR1, CDR2, and CDR3α, as well as CDR1, CDR2, and CDR3β pLDDTs, mean ipTM, TCR-pMHC-specific ipTM, iPSAE, and predicted DockQ scores (v1 and v2). Experimental vs. model structure comparison metrics include TCR-iRMSD, peptide iRMSD, MHC iRMSD, CDR3α RMSD, CDR3β RMSD, iRMSD, and lRMSD (blue), and DockQ scores (red) (* p-value < 0.001). (**B**) Distribution of AF3 model confidence metrics, including full complex, CDR1, CDR2, CDR3α, and CDR1, CDR2, CDR3β pLDDTs, mean ipTM, TCR-pMHC-specific ipTM, iPSAE, and predicted DockQ scores (v1 and v2). Distributions are stratified by quality tiers: Low Quality (LQ), Acceptable Quality (AQ), Medium Quality (MQ), and High Quality (HQ). Red dashed lines indicate acceptability thresholds; yellow dashed lines denote medium-quality thresholds; and green dashed lines indicate high-quality thresholds. **(C)** Mean precision and recall values of the training sets for each of the 265,720 combinations of confidence metrics and thresholds, evaluated using a five-fold cross-validation framework for each task: distinguishing LQ models from AQ/MQ/HQ, LQ/AQ from MQ/HQ, and LQ/AQ/MQ from HQ.

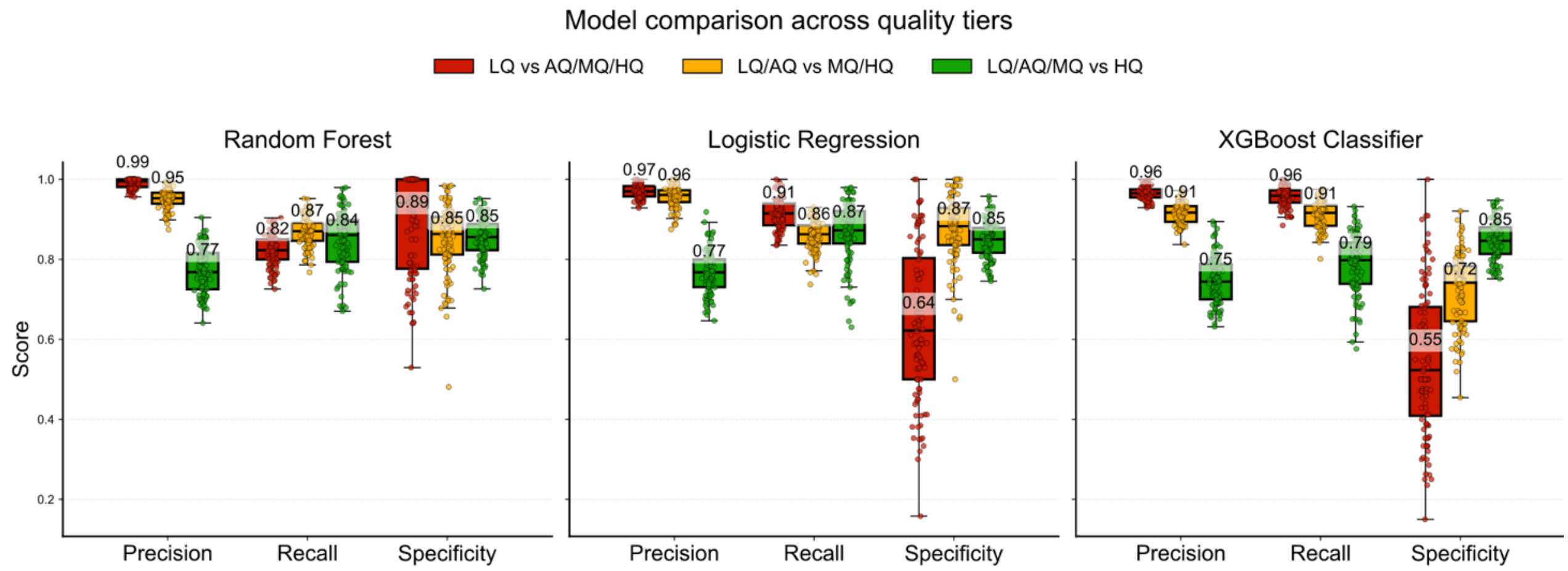

Supplementary Figure 5. Model comparison across quality tier classification tasks. Performance metrics (Precision, Recall, and Specificity) of Random Forest, Logistic Regression, and XGBoost Classifier across three different classification setups: Low Quality vs. rest (LQ vs rest; red), Low/Acceptable Quality vs. Medium/High Quality (LQ/AQ vs MQ/HQ; orange), and Low/Acceptable/Medium Quality vs. High Quality (LQ/AQ/MQ vs HQ; green). The classifiers were trained to distinguish quality tiers (LQ, AQ, MQ, and HQ) derived from experimental-versus-model structure comparisons using modelling confidence metrics. Boxplots and individual data points represent test set evaluations across 100 independent random seeds, with median values indicated above each plot.

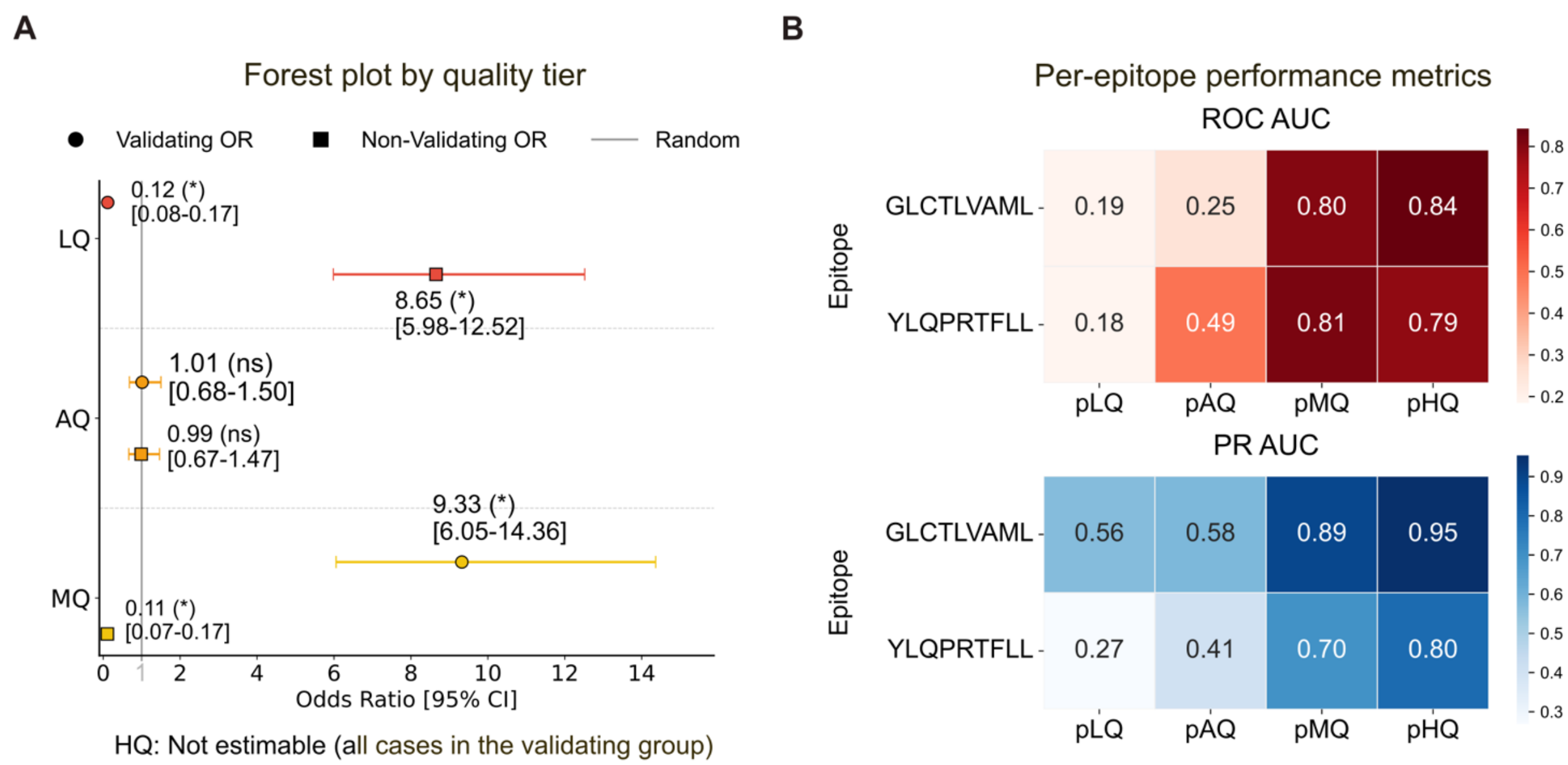

Supplementary Figure 6. Quality tier stratification identifies false positive TCR-pMHC interactions in TCRvdb. (**A**) Forest plots showing mean effect sizes (odds ratios) and 95% confidence interval for the association of: Low Quality (LQ), Acceptable Quality (AQ), Medium Quality (MQ) tiers with validating (circles) or non-validating (squares) TCR-pMHC complexes. Random expectation is indicated by a gray line (OR = 1) (* p-value<0.01). (**B**) Heatmap of ROC AUC and PR AUC values for Random Forest-predicted probabilities for each quality tier (LQ, AQ, MQ, HQ), stratified by epitope (GLCTLVAML and YLQPRTFLL).

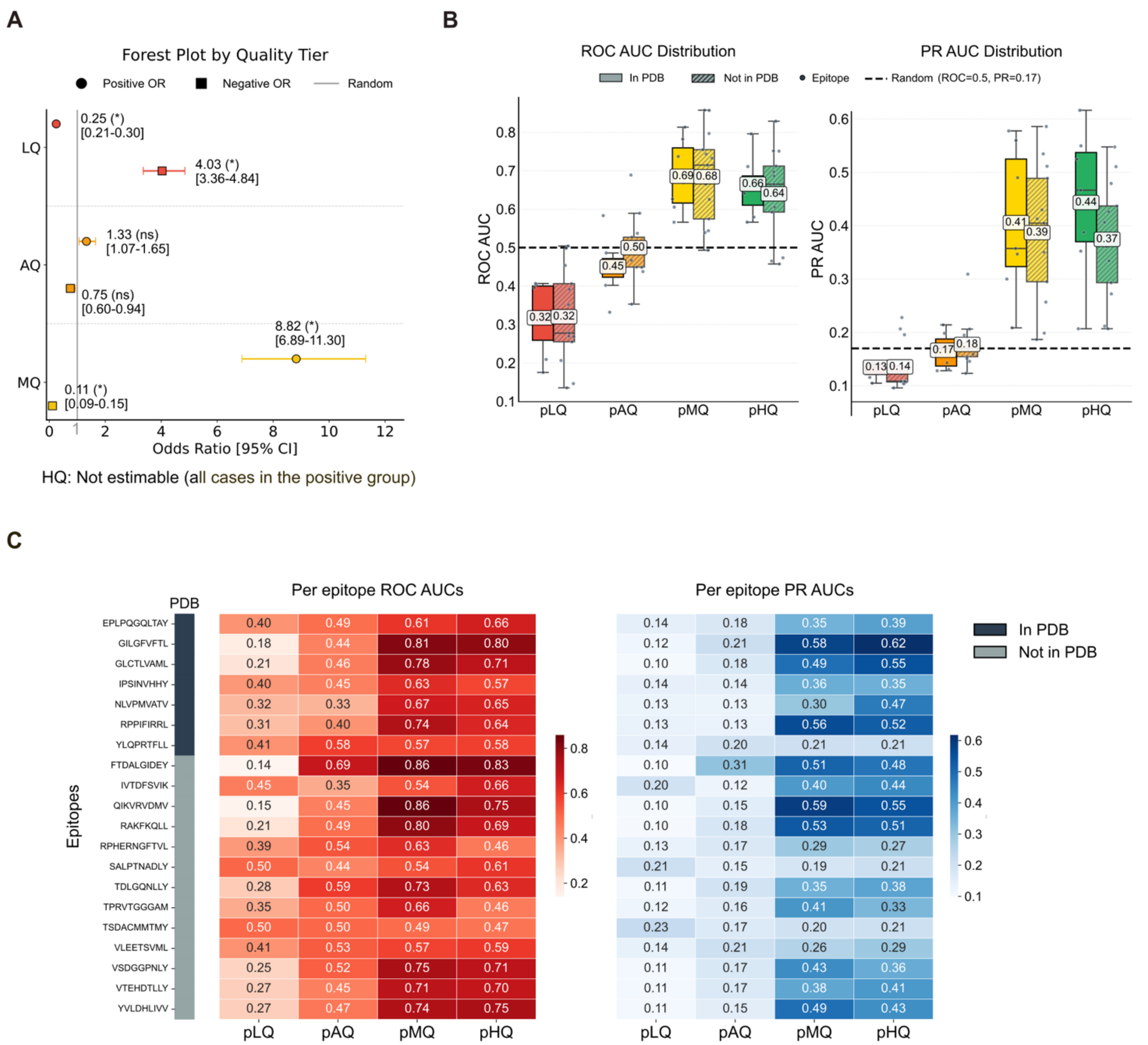

Supplementary Figure 7. Higher-quality structural models enrich for positive TCR-pMHC interactions in the IMMREP23 dataset. (**A**) Forest plots showing mean effect sizes (odds ratios) with 95% confidence intervals for the association of Low Quality (LQ), Acceptable Quality (AQ), Medium Quality (MQ) tiers with with positive (circles) or negative (squares) TCR-pMHC complexes. Random expectation is indicated by a gray line (OR = 1) (* p-value < 0.01). (**B**) Boxplots of ROC and PR AUCs for Random Forest-predicted probabilities for each quality tier (LQ, AQ, MQ, HQ) across individual epitopes in the IMMREP23 dataset. Each dot represents one epitope, split by whether the epitope is present in experimentally solved PDB complexes (Present in PDB: solid bars) or absent (Not in PDB: dashed bars). (**C**) Heatmap of ROC AUC and PR AUC values for Random Forest-predicted probabilities for each quality tier (LQ, AQ, MQ, HQ), stratified by epitope. Gray bars indicate whether the epitope is present (light gray) or absent (dark gray) in experimentally solved TCR-pMHC structures from the PDB.
